# Relationship between Young’s Modulus and Planar Density of Unit Cell, Super Cells (2 × 2 × 2), Symmetry Cells of Perovskite (CaTiO_3_) Lattice

**DOI:** 10.3390/ma14051258

**Published:** 2021-03-06

**Authors:** Marzieh Rabiei, Arvydas Palevicius, Sohrab Nasiri, Amir Dashti, Andrius Vilkauskas, Giedrius Janusas

**Affiliations:** 1 Faculty of Mechanical Engineering and Design, Kaunas University of Technology, LT-51424 Kaunas, Lithuania; marzieh.rabiei@ktu.edu (M.R.); andrius.vilkauskas@ktu.lt (A.V.); 2 Department of Materials Science and Engineering, Sharif University of Technology, Tehran 11365-9466, Iran; a.dashty@merc.ac.ir

**Keywords:** nano-perovskite (CaTiO_3_), X-ray diffraction, Young’s modulus, ultrasonic-pulse echo, planar density

## Abstract

Calcium titanate-CaTiO_3_ (perovskite) has been used in various industrial applications due to its dopant/doping mechanisms. Manipulation of defective grain boundaries in the structure of perovskite is essential to maximize mechanical properties and stability; therefore, the structure of perovskite has attracted attention, because without fully understanding the perovskite structure and diffracted planes, dopant/doping mechanisms cannot be understood. In this study, the areas and locations of atoms and diffracted planes were designed and investigated. In this research, the relationship between Young’s modulus and planar density of unit cell, super cells (2 × 2 × 2) and symmetry cells of nano CaTiO_3_ is investigated. Elastic constant, elastic compliance and Young’s modulus value were recorded with the ultrasonic pulse-echo technique. The results were C_11_ = 330.89 GPa, C_12_ = 93.03 GPa, C_44_ = 94.91 GPa and E = 153.87 GPa respectively. Young’s modulus values of CaTiO_3_ extracted by planar density were calculated 162.62 GPa, 151.71 GPa and 152.21 GPa for unit cell, super cells (2 × 2 × 2) and symmetry cells, respectively. Young’s modulus value extracted by planar density of symmetry cells was in good agreement with Young’s modulus value measured via ultrasonic pulse-echo.

## 1. Introduction

Perovskites have a general formula of ABO_3_. In these structures, the A site cation is a typical lanthanide, alkaline or alkaline-earth metal with 12-fold oxygen coordination, and the B-site is any one of a variety of transition metal cations [1]. Calcium titanate (CaTiO_3_) was established in 1839 by a Russian mineralogist Perovski, and materials with the same type of CaTiO_3_ were introduced as the perovskite structure. CaTiO_3_ has ionic bonds, as well as the ionic radii of Ca^2+^, O^2−^ and Ti^4+^ are 1 Å, 1.40 Å and 0.6 Å, respectively [2]. In recent years, researchers have focused on developing perovskites and their mechanical properties in order to obtain a high yield. Furthermore, CaTiO_3_ is a well-known component in ferroelectric perovskite category, which has been considerably utilized as a dopant/doping in electronic materials due to its dielectric manner and flexibility in structural transformations [3,4]. The modulus of elasticity (E) or Young’s modulus is defined as the proportion of the stress to the strain, created by the stress on the body when the body is in the elastic region [5]. The elastic constants are specified from the lattice crystal deformation against force. These elastic moduli are: Young’s modulus, shear modulus and volumetric modulus. These modules are registered via inherent elastic properties of materials and their resistance to deformation due to loading. Elastic behavior of materials is described by models such as Cauchy elastic, hypo-elastic and hyper-elastic. A hyper-elastic is a constitutive model for ideally elastic material that responds against stress gain from a strain energy density function, while for hypo-elastic material, their governing equation is independent of finite strain quantity except in the linearized state [6]. The elastic properties are intimately connected to the crystal structure, the intrinsic character of bonding between the atoms and the anisotropic nature of materials [7,8]; therefore, elastic constants can be derived from crystal lattice calculations [9]. There are several studies on the relationship between elastic constants and planes/directions in a lattice structure, for example, in [10,11,12]. One of the most accurate methods to measure the elastic stiffness constants and Young’s modulus is to determine the velocity of long-wavelength acoustic waves through the ultrasonic pulse-echo technique [13]. In a crystal structure, points, directions and planes are described with an indexing scheme, and planar density is obtained as the number of atoms per unit area, which are centered on a specific crystallographic plane with a defined index [14]. Since the discovery of X-rays at the end of the 19th century, this method has been often used for material characterization [15]. It is used to identify the atomic-scale structure of different materials in a variety of states [16]. X-ray diffraction is the only method that provides the specification of both the mechanical and microstructural character of each diffracted plane. These planes are used as a strain to quantify Young’s modulus in one or more planes/directions of the diffraction vector [17]. In forming, designing and manufacturing equipment industries, the use of non-destructive, accurate and convenient methods to determine the mechanical properties of materials is particularly important. Mechanical tests, such as tensile, strike and collision tests, are destructive. Ultrasonic methods are very time-consuming and require operator expertise in this area, and theoretical methods require time-consuming density functional theory (DFT) calculation and may need verification with experimental tests. Our proposed method only needs the XRD analysis, which is a routine test and calculation of planar density; therefore, it can be very significant in terms of industrial application. In this study, the effects of cell size on the accuracy of Young’s modulus calculation were considered. Locations of atoms and diffracted planes of unit cell, super cells (2 × 2 × 2) and symmetry cells of CaTiO_3_ are designed and investigated. The super cell is a cell that describes the same crystal but has a larger volume than a unit cell. By extension of a unit cell proportional to the lattice vectors, the super cells are generated. In super cells (2 × 2 × 2), the extension is twice of unit cell length in each direction; likewise, for super cells (8 × 8 × 8), the extension is 8 times. The result extracted by symmetry cells was in good agreement with results recorded via ultrasonic technique. Therefore, this new approach of exploration of reliable Young’s modulus quantity based on XRD is proposed for either single crystal or polycrystalline of CaTiO_3_.

## 2. Experimental

### 2.1. Materials

In this study, for synthesis CaTiO_3_, titanium (IV) butoxide, calcium chloride dehydrate, sodium hydroxide and ethanol reagents were purchased from Sigma Aldrich (Taufkirchen, Germany) and deionized water as the solvent for dispersions was prepared.

### 2.2. Instrumentation

In this research, a Bruker D8 Advance X-ray diffractometer (Kaunas, Lithuania) with ${\mathrm{Cu}}_{K}{}_{\alpha }$ radiation was used. The powder X-ray diffraction was taken at 40 kV and 40 mA, and it was registered at a scanning rate of 2.5 degrees/minute and a step size of 0.02 degrees. The XRD peaks were interpreted by High Score X’Pert software (4.9.0) analysis to get the output ASC type files. The pulse-echo technique was applied for the determination of sound velocity for both transverse and longitudinal ultrasonic signals. For ultrasonic measurement, the model of pulser receiver and oscilloscope were Panametrics Co. (waltham, MA, USA) and Iwatsu (Tokyo, Japan) (100 MHz), respectively. For powder pressing, the model of mechanical machines was CD04-Z and CIP (CP 360). Additionally, the specific surface area of the sample was investigated by desorption isotherms of nitrogen (N_2_) gas via using a Brunauer-Emmett-Teller (BET) apparatus Gemini V analyzer, micrometrics GmbH (Tehran, Iran). Moreover, transmission electron microscopy (TEM) CM 10-Philips (Tehran, Iran) with acceleration voltage from 50 to 80 KV was utilized.

### 2.3. Methods

#### 2.3.1. Synthesis of Nano-Powder CaTiO_3_

Calcium titanate (CaTiO_3_) was synthesized by solvothermal method. A simple procedure, namely the solvothermal method, was performed for the synthesis of CaTiO_3_ (Figure S1). In the first step, (1) calcium chloride dehydrate was stirred with ethanol and deionized water. (2) Titanium (IV) butoxide and ethanol were added to the system drop by drop, under stirring for around 10 min (750 rpm). The molar ratio of ingredients was achieved to calcium chloride dehydrate = 1, ethanol = 5, Titanium (IV) butoxide = 1 and deionized water = 100 respectively. (3) To create pH = 14, sodium hydroxide solution was utilized. (4) The produced solution was placed into the autoclave and the temperature was ~250 °C for 5 h. (5) Afterward, the product was under the drying conditions involved at 110 °C and 0.76 bar, respectively. (6) After a day, the mixture was washed, (7) filtered and dried (110 °C for 4 h), respectively. This method was used in previous studies [18,19].

#### 2.3.2. X-ray Diffraction of CaTiO_3_ and Planar Density Calculations

Combining X-ray diffraction of crystalline CaTiO_3_ and calculation of planar density values of each diffracted plane was performed. In our study, the atomic density of each plane was considered as the planar density, which was determined as the area of atoms with the center positioned at the plane divided by the total area of the plane, and it is a determinant factor for mechanical properties of each plane. Planar density is a unitless parameter, and its value is less than 1 in each cell. Furthermore, the values of planar density are related to the positions and situations of atoms in the planes. For determination of atomic area, the Crystal Maker, Version 10.2.2 software was performed. First of all, the three-dimensional (3D) geometry of crystal structures was designed, and then, from the intersection area of each diffracted plane with atoms located at the plane, the atomic area was calculated. When an atom with diameter D was involved completely, the atomic area will be $\;A=\pi {\left(\frac{D}{2}\right)}^{2}$; otherwise, it will be a percentage of this amount.

#### 2.3.3. Ultrasonic Pulse-Echo Technique of CaTiO_3_

An ultrasonic wave is a type of elastic wave spread in the medium with high frequency to obtain the Young’s modulus value of samples. Mastering the ultrasonic parameters can be used to acquire more accurate values of mechanical properties [20]. Recently, different studies on mechanical properties have been done by ultrasonic techniques. Basically, the crossing of longitudinal and transverse waves in nano- or microstructures is performed at different velocities. Each returned velocity is considered as the represented properties.

**Figure 1:**
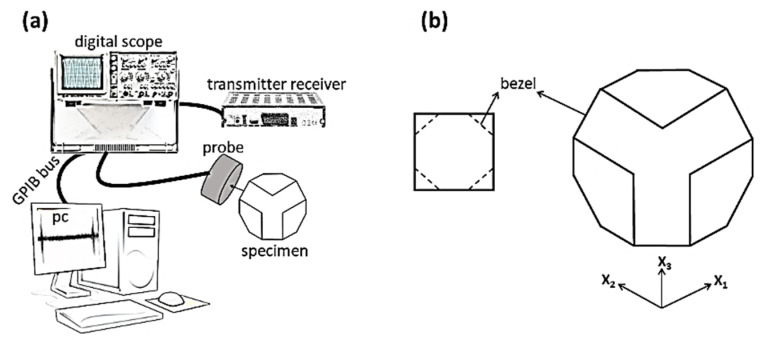
Schematic of (**a**) ultrasonic pulse instrument and (**b**) a sketch of prepared CaTiO_3_ sample.

For ultrasonic measurements based on the Christoffel procedure, the first cubic specimen of CaTiO_3_ was prepared by cold isostatic press. The schematics of ultrasonic measurement are depicted in Figure 1a. The main part of the ultrasonic system is the pulser-receiver, which creates an electric pulse and stimulates the probe. Furthermore, the produced pulses enter the specimen, and after a sweep, they can be received via a probe. In this measurement, some drops of water were utilized to prevent the depreciation of waves in the air, and the effect of hand pressure on the probe was decreased [21].

At any position in the sample, a local coordinate is adjusted, such as X_1_, the radial coordinate; X_2_, the circumferential coordinate; and X_3,_ the axial coordinate. V_i/j_ denotes the velocity of an ultrasound wave propagating in the X_i_ direction with particle displacements in the X_j_ direction. V_i/j_ with the same i and j is longitudinal, and with i ≠ j is related to the transverse waves. For the measurement of quasi-longitudinal or quasi-transverse velocity (V_ij/ij_), the specimen should be cut (bezel) on the edges of the surfaces perpendicular to the X directions. A sketch of the sample is represented in Figure 1b.

## 3. Results

### 3.1. X-ray Diffraction of CaTiO_3_ and Planar Density Calculations

**Figure 2:**
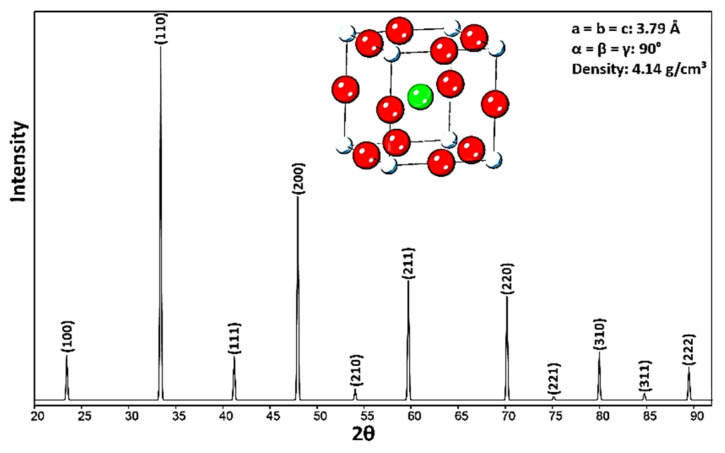
X-ray diffraction of CaTiO_3_ (powder sample).

The XRD pattern of CaTiO_3_ is presented in Figure 2. The characteristic peaks of CaTiO_3_ correspond to the report in Ref [22]. The crystal structure of CaTiO_3_ is cubic, the atomic positions of Ti are at (000), Ca at ($\frac{1}{2},\frac{1}{2},\frac{1}{2}$) and O at ($\frac{1}{2},0,0$), ($0,\frac{1}{2},0$), ($0,0,\frac{1}{2}$). According to X-ray powder diffraction results, the lattice parameter is 3.79 ± 0.02 Å, which is in good corresponds with the amount recorded in the Ref [23]. In addition, crystallographic parameters (Table S1) of CaTiO_3_ and analyzed data by X’Pert [24] nasiri are recorded as the cell volume = 54.44 ± 0.01 Å^3^ and crystal density = 4.14 ± 0.01 g/cm^3^, and the space group is Pm-3m. In addition, the crystal size of CaTiO_3_ was calculated by the Monshi–Scherrer equation (Figure S2) [25] and BET analysis. The crystal size values were registered at ~59.10 and 63.02 nm, respectively. The Monshi–Scherrer method is described in Section 2 of the supporting information. Furthermore, a TEM image of CaTiO_3_ is shown in Figure S3. According to the images shown in Figure S3, the size of CaTiO_3_ particles basically corresponds to the crystallite size, and it is clear that particles of powder have nanoscale and size can be reported almost ±50 nm.

**Figure 3:**
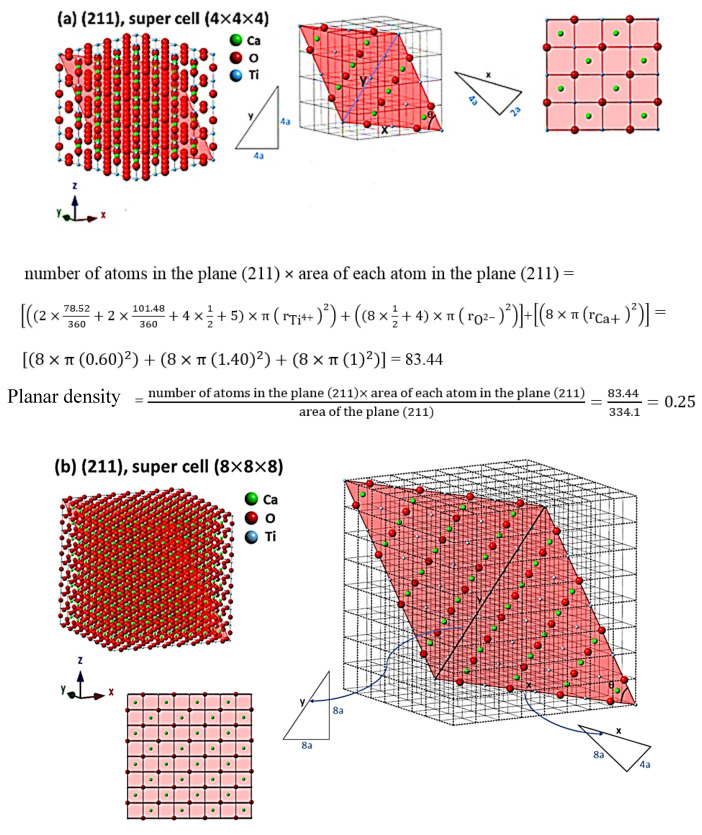

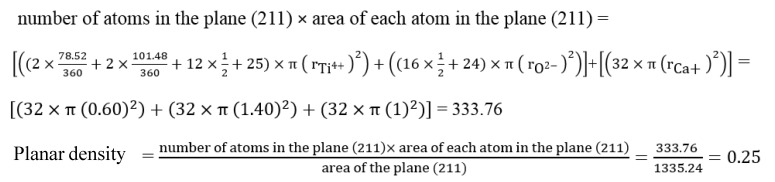
Geometry of planes and calculations of planar density of (**a**) (211) super cell (4 × 4 × 4) and (**b**) (211) super cell (8 × 8 × 8) (which shows and emphasizes the symmetry of (8 × 8 × 8) super cells).

**Figure 4:**
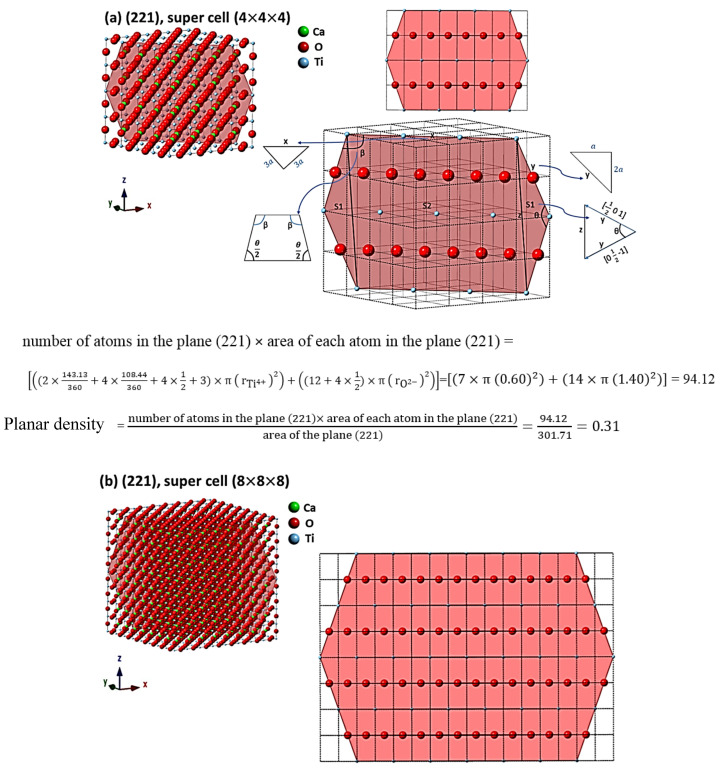

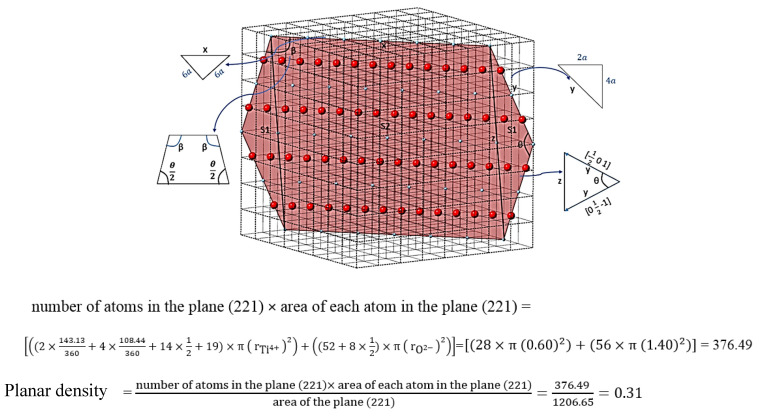
Geometry of planes and calculations of planar density of (**a**) (221) super cell (4 × 4 × 4) and (**b**) (221) super cell (8 × 8 × 8) (which shows and emphasizes the symmetry of (8 × 8 × 8) super cells).

**Figure 5:**
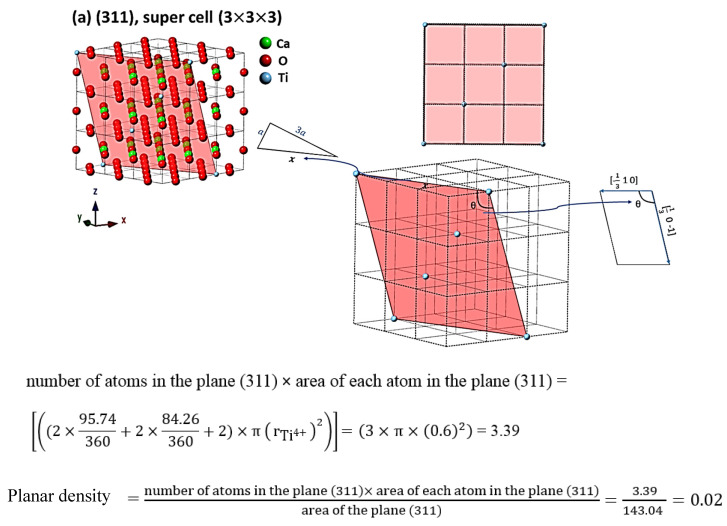

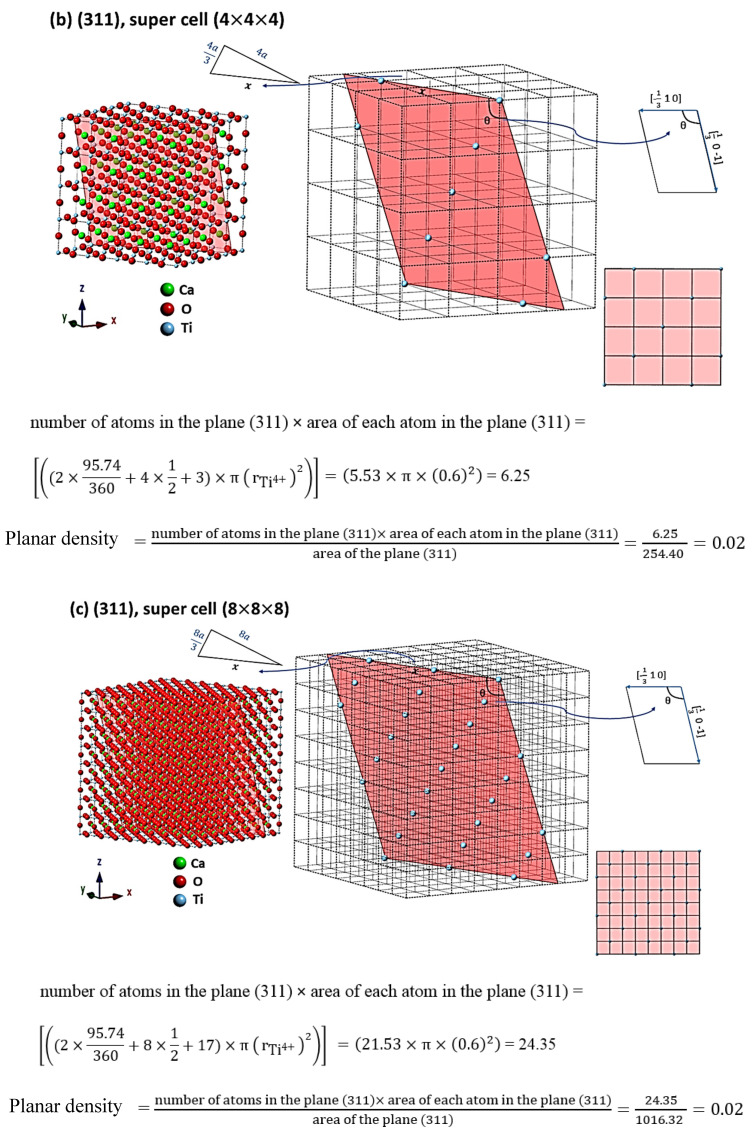
The concept of a symmetry cell; geometry of planes and calculations of planar density of (**a**) (311) super cell (3 × 3 × 3), (**b**) (311) super cell (4 × 4 × 4) and (**c**) (311) super cell (8 × 8 × 8).

**Figure 6:**
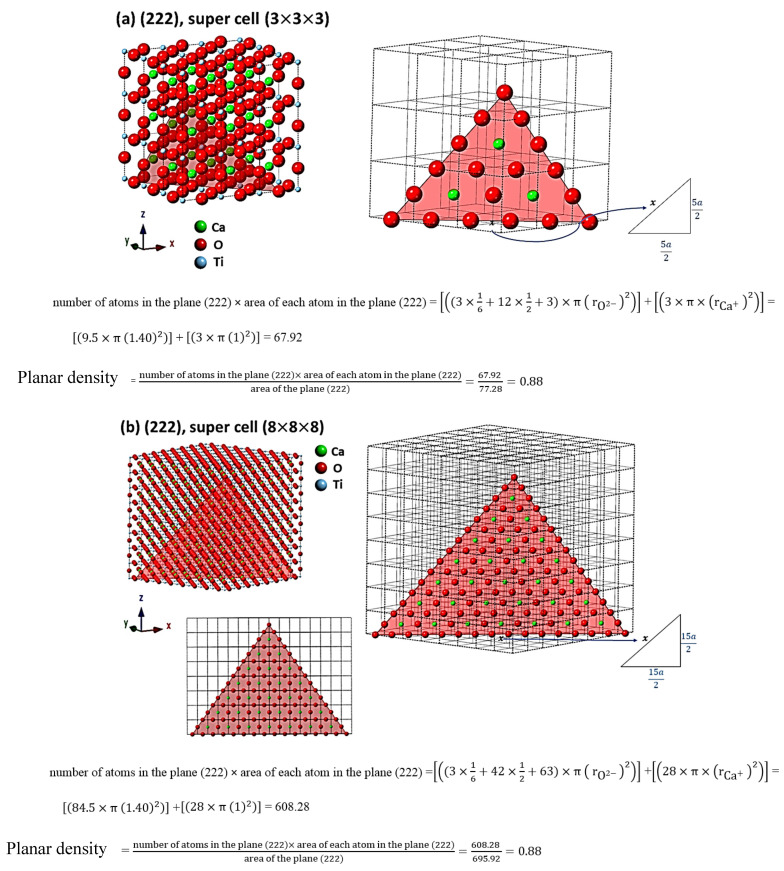
Geometry of planes and calculations of planar density of (**a**) (222) super cell (3 × 3 × 3) and (**b**) (222) super cell (8 × 8 × 8).

For the evaluation of cells as the results, the comprehensive calculations of the planar density of diffracted planes in the unit cell, super cells (2 × 2 × 2) and super cells (8 × 8 × 8) of CaTiO_3_ lattice are presented in Figures S4–S6 respectively. In addition, the locations of atoms, geometry of planes and calculations of planar density of (211) super cell (4 × 4 × 4), (211) super cell (8 × 8 × 8), (221) super cell (4 × 4 × 4), (221) super cell (8 × 8 × 8), (311) super cell (3 × 3 × 3), (311) super cell (4 × 4 × 4), (311) super cell (8 × 8 × 8), (222) super cell (3 × 3 × 3) and (222) super cell (8 × 8 × 8) are depicted briefly in Figure 3, Figure 4, Figure 5 and Figure 6 respectively. Furthermore, the completed calculations with their figures are shown in Figures S7–S10.

### 3.2. Investigation of Results Obtained from Ultrasonic Pulse-Echo Technique of CaTiO_3_

Taking into account the Christoffel equation, the connection between ultrasonic phase velocity and the stiffness matrix is given as follows:

$$(C_{\mathrm{ijkl}}l_{j}l_{l}-{\rho V}^{2}\delta_{\mathrm{ik}})\alpha_{k}\;=\;0$$

where V is the ultrasonic phase velocity, $C_{\mathrm{ijkl}}$ is the general stiffness matrix, ρ is the material density, l is the orientation of propagation, $\alpha_{k}$ is the polarization direction and $\delta_{\mathrm{ik}}$ is the Kronecker delta (note that i, j, k, I = 1 to 3). For the extraction and calculation of elastic constants from ultrasonic measurements based on the Christoffel equation, with the propagation in X_1_, X_2_ and X_3_ directions, all of the diagonal elements of the stiffness matrix are obtained. For the determination of whole constants, we cut the specimen on the edges of the surfaces perpendicular to principal directions (bezel) and the velocity was measured from the propagation of ultrasound wave normal to these planes.

Based on Equations (1)–(5) [26,27] and the measured velocity according to the Table 1, stiffness constants values were obtained. $C_{11}$ is in the agreement with longitudinal distortion and longitudinal compression/tension, so $C_{11}$ can be described as the hardness. Moreover, the transverse distortion is connected to the $C_{12}$, and $C_{12}$ is obtained from the transverse expansion correlated to the Poisson’s ratio. $C_{44}$ is based on the shear modulus, as well as $C_{44}$ is in the settlement with $C_{11}$ and $C_{12}\;$ [26].

(1) C11 = ρV112

(2) C22 = ρV222

(3) C66 = ρV122= ρV212

(4) C12 = (C11+C66−2ρV12122)(C22+C66−2ρV12122) − C66

(5) C44 = ρV232= ρV322

**Figure 7:**
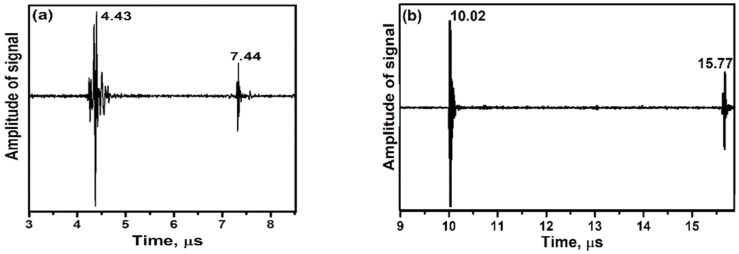
Recorded signals extracted via (**a**) longitudinal waves and (**b**) transverse waves of CaTiO_3_ specimen.

After substitution and calculation, C_11_, C_12_ and C_44_ were registered at 330.89, 93.03 and 94.91 GPa respectively. These values of CaTiO_3_ were in good agreement with the values submitted in the [28,29,30]. Moreover, with the ultrasonic technique, longitudinal and transverse waves can be utilized for determining Young’s modulus quantity [31,32]. The longitudinal and transverse waves of CaTiO_3_ sample are shown in Figure 7. In this method, by measuring the waves velocity and density of specimen, the determination of Young’s modulus quantity was carried out (Equation (6)).

(6) $$E\;=\;\frac{4\rho {\left(\frac{L}{t_{s}}\right)}^{2}\left(3t_{s}^{2}-4t_{l}^{2}\right)}{t_{s}^{2}-t_{l}^{2}}$$

where, $t_{s}$ and $t_{l}$ are differences between two echo in longitudinal and transverse waves, respectively [33,34]. According to the results shown in Figure 7, $t_{s}$ and $t_{l}$ values are calculated as 5.75 and 3.01 μs, respectively. In addition, the density of the specimen is recorded as 3857.30 $\frac{\mathrm{Kg}}{m^{3}}$, and the length of the specimen after powder pressing reached 11.21 mm. After calculation, Young’s modulus value of CaTiO_3_ was 153.87 GPa. This value corresponds with the value reported by Ramajo et al. [35].

### 3.3. Calculations: Relationship between Elastic Stiffness-Compliance Constants, Young’s Modulus and Planar Density Extracted through the Unit Cell, Super Cells (2 × 2 × 2) and Symmetry Cells of CaTiO_3_ Lattice

Three elastic constants of CaTiO_3_ were calculated via the ultrasonic technique. For the cubic CaTiO_3_ system, the relationship between stiffness ($C_{\mathrm{ij}}$) and compliance constant ($S_{\mathrm{ij}}$) are provided in Equations (7)–(9) [27,36]. The values resulted via Equations (7)–(9) are 0.0034, −0.0007 and 0.0105 GPa for $S_{11}$, $S_{12}$ and $S_{44}$, respectively. Furthermore, Young’s modulus of each diffracted plane of CaTiO_3_ can be written as Equation (10) [37].

(7) $$S_{11}=\;\frac{C_{11}+C_{12}}{\left(C_{11}-C_{12}\right)\left(C_{11}+2C_{12}\right)}$$

(8) $$S_{12}=\;\frac{-C_{12}}{\left(C_{11}-C_{12}\right)\left(C_{11}+2C_{12}\right)}$$

(9) $$S_{44}=\;\frac{1}{C_{44}}$$

(10) $$\frac{1}{E_{hkl}}\;=\;S_{11}-\;2\left[\left(S_{11}-S_{12}\right)-\frac{1}{2}S_{44}\right]\left[\frac{h^{2}k^{2}+\;k^{2}l^{2}+\;l^{2}h^{2}}{\left(h^{2}+\;k^{2}+\;l^{2}\right)}\right]$$

The planar density and Young’s modulus values related to the each diffracted plane of the unit, super (2 × 2 × 2), symmetry and super (8 × 8 × 8) cells of CaTiO_3_ lattice are tabulated in Table 2.

## 4. Discussion

According to Table 2 and Figure 3, Figure 4, Figure 5 and Figure 6, the expanded cells have an optimum matrix, and in this case, achieving the optimum matrix is introduced as the symmetry cells. An optimum matrix is the minimum extension for a specific plane of the unit cell to a super cell from which the density plane of that plane does not change. For example, symmetry cell (optimum matrix) of (311) plane is (3 × 3 × 3), which means that after extending to a greater matrix such as (4 × 4 × 4) or (8 × 8 × 8), planar density values will be similar (Figure 5a–c). Real planar density values of each plane are obtained from the symmetry cell, because once the symmetry of each plane is reached, with the extension of that plane to infinity (real plane), the planar density does not change. In addition, to recognize the symmetry cell, knowing some parameters such as crystal lattice, locations of atoms in the planes and index of planes is essential. Therefore, to determine Young’s modulus values based on the planar density of CaTiO_3_, the symmetry cells should be found. It is very interesting that symmetrical or real values are related to the super cells of the (8 × 8 × 8) matrix, because in matrix (8 × 8 × 8), lattice correspondence in all directions is available; therefore, real planar density values should be calculated for the super cell of (8 × 8 × 8) matrix. To confirm this, calculations of real planar density and geometry of atoms and planes of (211), (221), (311) and (222) in super cells (8 × 8 × 8) are presented in Figure 3b, Figure 4b, Figure 5c and Figure 6b, respectively. It is clear that finding the exact situation of planes and geometries is sophisticated, but with when they are known, the results obtained from Young’s modulus values will have fewer errors. The basic supposition is that when the planar density is raised, the motion of atoms with the mechanism of dislocation movement needs high forces. Dislocations are regions in the lattice where an additional plane of atoms have been included abstracted from an ideal crystal (without imperfections). Dislocations are caused by losing acoustic energy, and this matter will affect the values of wavelength and time of ultrasonic waves [38].

The force (W), which is needed for the movement of atoms in each plane, is obtained from Equation (11) [39].

(11) $$W\;=\;\frac{E}{2\left(1+\nu \right)}\;b^{2}l$$

In Equation (11), E is Young’s modulus, b is Burgers vector, l is dislocation length and ν is Poisson’s ratio. The higher value of force is in accordance with the modulus of elasticity (Young’s modulus), which would be higher.

**Figure 8:**
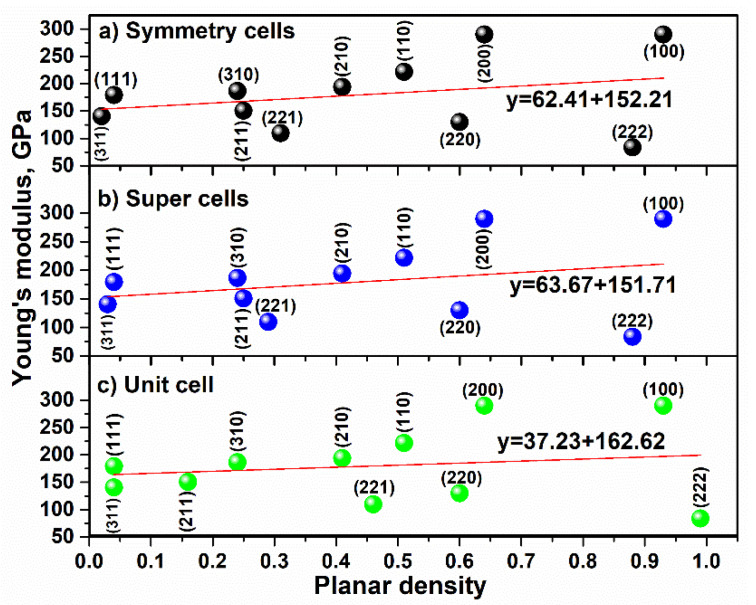
Young’s modulus versus planar density values of each diffracted plane related to the (**a**) symmetry cells, (**b**) super cells (2 × 2 × 2) and (**c**) unit cell of CaTiO_3_.

To compare Young’s modulus values of CaTiO_3_ in a unit cell, super cells (2 × 2 × 2) and symmetry cells, the fitting of Young’s modulus values extracted by each diffracted plane versus planar density values is presented in Figure 8. According to the results (shown in the Figure 8) and the straight fitting line, Young’s modulus values of unit cell, super cells (2 × 2 × 2) and symmetry cells were calculated as 162.62 ± 0.4 GPa, 151.71 ± 0.4 GPa and 152.21 ± 0.4 GPa, respectively. As expected, the Young’s modulus value of symmetry cells of CaTiO_3_ (152.21 ± 0.4 GPa) is in good agreement with experimental Young’s modulus value extracted via ultrasonic-echo technique (153.87 ± 0.2 GPa). Moreover, Young’s modulus value of unit cell (162.62 ± 0.4 GPa) has a greater difference with experimental Young’s modulus value, and as a result, the unit cell of CaTiO_3_ cannot be represented as whole cells. This is because in a unit cell of CaTiO_3_, crystalline defects are not considered and is especially controlling of deformation, and displacement of atoms in the planes is related to the dislocation networks [40]. Further, a unit cell of CaTiO_3_ is not involved in imperfections (such as dislocations, Frenkel defect and Schottky defect) with respect to the super cell [41]; therefore, the slope line value of the unit cell is reported (37.23) to be less than the slope line value of super cells (2 × 2 × 2) (63.67) and symmetry cells (62.41). Consequently, the effect of imperfections in expanded cells (super cells) is very impressive, so the unit cell of CaTiO_3_ is considered as the ideal lattice, while symmetry cells such as (8 × 8 × 8) of CaTiO_3_ are real lattices [42]; this is consistent with the experimental Young’s modulus. It is clear that each imperfection will be caused by a decreasing Young’s modulus [43], and in Figure 8, this matter is confirmed when the Young’s modulus value (intercept) in the unit cell of CaTiO_3_ is higher than in super cells (2 × 2 × 2) and symmetry cells. Apparently, a unit cell of CaTiO_3_ is represented by the volume of a real crystal, so the unit cell is useful to acquire theoretical density. Nevertheless, calculations of planar density based on the unit cell were obtained, but with errors.

## 5. Conclusions

1. CaTiO_3_ as a category of perovskite is successfully synthesized via solvothermal method.
2. Crystal size values of CaTiO_3_ are calculated as ~59.10 and 63.02 through the Monshi-Scherrer method and BET analysis, and the crystal size values were confirmed by TEM image.
3. Planar density is responsible for modulus of elasticity of that plane; therefore, for the first time, comprehensive calculations of geometry, location and planar density values of CaTiO_3_ were shown.
4. Elastic stiffness constants and Young’s modulus values of CaTiO_3_ were obtained by ultrasonic-echo method (C_11_ = 330.89, C_12_ = 93.03, C_44_ = 94.91 GPa and E = 153.87 ± 0.2 GPa).
5. Young’s modulus values of CaTiO_3_ extracted by planar density and least square method were calculated as 162.62 ± 0.4, 151.71 ± 0.4 and 152.21 ± 0.4 GPa for unit cell, super cells (2 × 2 × 2) and symmetry cells, respectively.
6. The Young’s modulus value of CaTiO_3_ reported by symmetry cells is in good agreement with Young’s modulus value reported by ultrasonic-echo technique and the literatre.
7. A unit cell of CaTiO_3_ is not representative of the distribution of atoms on the planes; therefore, to obtain the real value of planar density and find the symmetry of distribution of atoms on the planes, expanded cells and utilizing symmetry cells are suggested.
8. Obtaining the planar density values based on unit cell or each super cells except for (8 × 8 × 8) is an estimation.
9. The real value of Young’s modulus of CaTiO_3_ should be extracted by symmetry cells or super cells (8 × 8 × 8).
10. The value of Young’s modulus of CaTiO_3_ extracted with this method can be applied for industrial applications.

## Figures and Tables

**Table 1:** The values of longitudinal and transverse velocity of the sample.

Longitudinal Velocity (m/s)	Transverse Velocity (m/s)	Quasi Longitudinal or Quasi Transverse (m/s)
V_1/1_ = 9261.85	V_2/3_ = 4960.5	V_12/12_ = 4976.63
V_2/2_ = 8013.51	V_1/2_ = 4283.65	

**Table 2:** Planar density and Young’s modulus values of the unit cell, super cells (2 × 2 × 2) and symmetry cells of CaTiO_3_.

Index	Planar Density of Unit Cell	Planar Density of Super Cell (2 × 2 × 2)	Planar Density of Symmetry Cells	Planar Density of Super Cell (8 × 8 × 8)	Young’s Modulus (GPa)
(100)	0.93	0.93	0.93 in (2 × 2 × 2)	0.93	290.059
(110)	0.51	0.51	0.51 in (2 × 2 × 2)	0.51	221.652
(111)	0.04	0.04	0.04 in (2 × 2 × 2)	0.04	179.354
(200)	0.64	0.64	0.64 in (2 × 2 × 2)	0.64	290.059
(210)	0.41	0.41	0.41 in (2 × 2 × 2)	0.41	194.176
(211)	0.16	0.25	0.25 in (2 × 2 × 2)	0.25	150.612
(220)	0.6	0.6	0.6 in (2 × 2 × 2)	0.6	129.810
(221)	0.46	0.29	0.31 in (4 × 4 × 4)	0.31	109.622
(310)	0.24	0.24	0.23 in (4 × 4 × 4)	0.23	186.471
(311)	0.04	0.03	0.02 in (3 × 3 × 3)	0.02	140.386
(222)	0.99	0.88	0.88 in (3 × 3 × 3)	0.88	83.615

## Data Availability

Data supporting the findings of this study are available from the corresponding author upon request.

## Supplementary Materials

The following are available online at https://www.mdpi.com/1996-1944/14/5/1258/s1, Figure S1: Synthesis route of CaTiO_3_, Table S1: Crystallographic parameters of each individual XRD pattern related to the CaTiO_3_, Figure S2: Linear plot of modified Scherrer equation related to the CaTiO_3_, Figure S3: TEM image of CaTiO_3_ powder, Figure S4: Geometry of planes and calculations of planar density of (a) (100), (b) (110), (c) (111), (d) (200), (e) (210), (f) (211), (g) (220), (h) (221), (i) (310), (j) (311) and (k) (222) related to the unit cell of CaTiO_3_, Figure S5: Geometry of planes and calculations of planar density of (a) (100), (b) (110), (c) (111), (d) (200), (e) (210), (f) (211), (g) (220), (h) (221), (i) (310), (j) (311) and (k) (222) related to the super cells (2 × 2 × 2) of CaTiO_3_, Figure S6: Geometry of planes and calculations of planar density of (a) (100), (b) (110), (c) (111), (d) (200), (e) (210), (f) (220), (g) (310) (4 × 4 × 4) and (h) (310) (8 × 8 × 8) related to the super cells (8 × 8 × 8) of CaTiO_3_, Figure S7: Geometry of planes and calculations of planar density of (a) (211) super cell (4 × 4 × 4) and (b) (211) super cell (8 × 8 × 8), Figure S8: Geometry of planes and calculations of planar density of (a) (221) super cell (4 × 4 × 4) and (b) (221) super cell (8 × 8 × 8), Figure S9: Geometry of planes and calculations of planar density of (a) (311) super cell (3 × 3 × 3), (b) (311) super cell (4 × 4 × 4) and (c) (311) super cell (8 × 8 × 8), Figure S10: Geometry of planes and calculations of planar density of (a) (222) super cell (3 × 3 × 3), (b) (222) super cell (8 × 8 × 8).
