# Supplementary material for: Relationship between Young’s Modulus and Planar Density of Unit Cell, Super Cells (2 × 2 × 2), Symmetry Cells of Perovskite (CaTiO3) Lattice
Source: Materials (Basel). 2021 Mar 6;14(5):1258. doi: 10.3390/ma14051258 (PMC7961767; doi:10.3390/ma14051258)
Supplement: Supplementary file 1 [file materials-14-01258-s001.pdf]

materials

Supplementary Materials

Relationship between Young's Modulus and Planar Density of Unit Cell, Super Cells (2×2×2), Symmetry Cells of Perovskite ($CaTiO_3$) Lattice

Marzieh Rabiei, Arvydas Palevicius, Sohrab Nasiri, Amir Dashti, Andrius Vilkauskas and Giedrius Janusas

1. Experimental

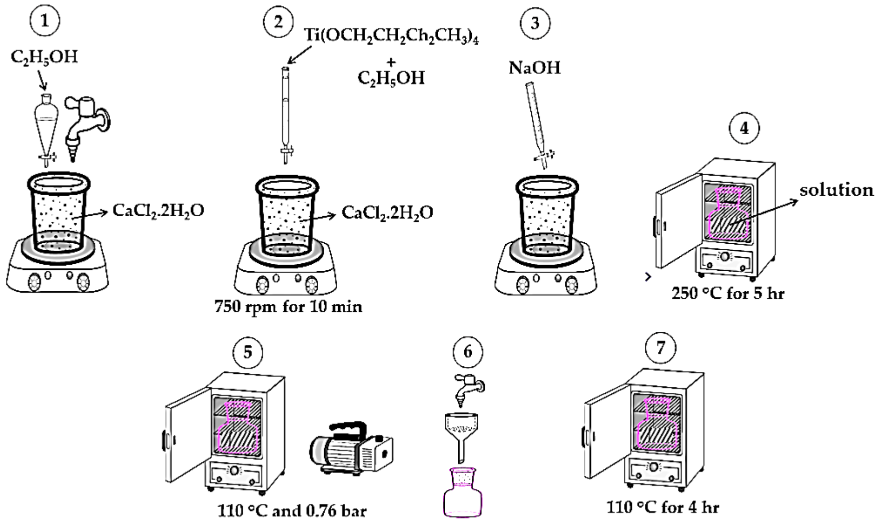

Figure S1. Synthesis route of $CaTiO_3$.

2. Crystalline Size Calculation with Modified Scherrer Equation

According to the Monshi-Scherrer equation, for finding the size of the crystal, **Equation (S1)** is utilized. The modified scherrer equation can provide the advantage of decreasing errors to give a more accurate value of L from all or some of the different peaks [1].

$$\mathrm{Ln}\beta = \mathrm{Ln}\left(\frac{K\lambda}{L}\right) + \mathrm{Ln}\left(\frac{1}{\mathrm{Cos}\theta}\right) \quad \text{(S1)}$$

The linear plot of Ln β (β in radians) versus Ln $\left(\frac{1}{\mathrm{Cos}\theta}\right)$ (degree) can be a linear plot for all peaks, the least squares statistical method is used to decrease the sources of errors. After stablishing the most accurate linear plot, the value of $\mathrm{Ln}\left(\frac{K\lambda}{L}\right)$ can be obtained from the intercept. The $e^{(\text{intercept})}$ gives $\left(\frac{K\lambda}{L}\right)$, single value of L is obtained from all of the available peaks. Ln β versus $\mathrm{Ln}\left(\frac{1}{\mathrm{Cos}\theta}\right)$ is depicted in the plots of **Figure S2,** with the equations of the linear least squares method obtained from the linear regression of data in plot. After plotting and obtaining the linear equation for the least squares method of peaks, **Equation (S2)** is done. The crystallographic values of each individual XRD pattern of $CaTiO_3$ are tabulated in **Table S1**.

$$\left(\frac{K\lambda}{L}\right) = e^{(\text{intercept})} \quad \text{(S2)}$$

Linear equation of calcium titanate was recorded by y = 1.4941x - 6.0682 with intercept value of - 6.0682. Nevertheless, the intercept was calculated as $e^{(-6.0682)}$ = 0.00232. After the calculation, the value of crystal size achieved to ~ 59.10 nm. (K is the shape factor, usually taken as 0.89 for ceramic materials and λ is wavelength of radiation in nanometer ($\lambda_{Cuk\alpha}$=0.15405 nm)). Base of this method is related to the logarithmic function, and logarithmic function can decrease the errors of crystal size calculations [2]. The BET specific surface area of $CaTiO_3$ sample is ~ 24.68 m^2/g. Furthermore, the value of crystal size extracted by BET is achieved to 63.02 nm. As a result, the value of crystalline size calculated from X-Ray diffraction was in the well correspondence with value extracted from BET.

Table S1. Crystallographic parameters of each individual XRD pattern related to the $CaTiO_3$.

$CaTiO_3$										
**2θ (degree)**	**β=FWHM (degree)**	**θ (degree)**	**cosθ (degree)**	**1/cosθ (degree)**	**Ln(1/cosθ) (degree)**	**β=FWHM (radian)**	**Ln β (radian)**	**4 sinθ (degree)**	**β(radian).cosθ (degree)**	**hkl**
23.45	0.128	11.73	0.98	1.0213	0.02109	0.00223	-6.07484	0.81	0.00219	100
33.41	0.136	16.71	0.96	1.04409	0.04315	0.00237	-6.04332	1.15	0.00227	110
41.22	0.145	20.61	0.94	1.06837	0.06614	0.00253	-5.97924	1.41	0.00237	111
47.97	0.154	23.98	0.91	1.09451	0.09030	0.00268	-5.91902	1.63	0.00243	200
54.06	0.163	27.03	0.89	1.12263	0.11567	0.00284	-5.86223	1.81	0.00252	210
59.71	0.185	29.85	0.86	1.15301	0.14238	0.00322	-5.73562	1.99	0.00277	211
70.18	0.165	35.09	0.82	1.22212	0.20058	0.00288	-5.85003	2.29	0.00236	220
75.14	0.191	37.57	0.79	1.26166	0.23242	0.00333	-5.70370	2.44	0.00263	221
79.98	0.217	39.99	0.77	1.30521	0.26636	0.00378	-5.57608	2.57	0.00291	310
84.86	0.180	42.43	0.74	1.35483	0.30367	0.00314	-5.76302	2.69	0.00232	311
89.50	0.230	44.75	0.71	1.40808	0.34222	0.00401	-5.51790	2.81	0.00284	222

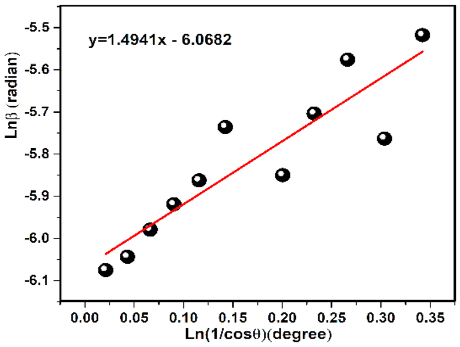

Figure S2. Linear plot of modified Scherrer equation related to the $CaTiO_3$.

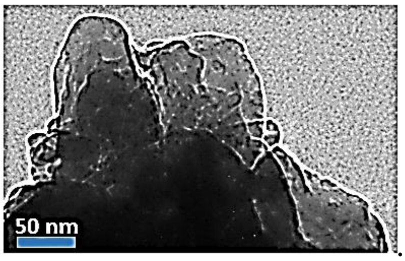

Figure S3. TEM image of $CaTiO_3$ powder.

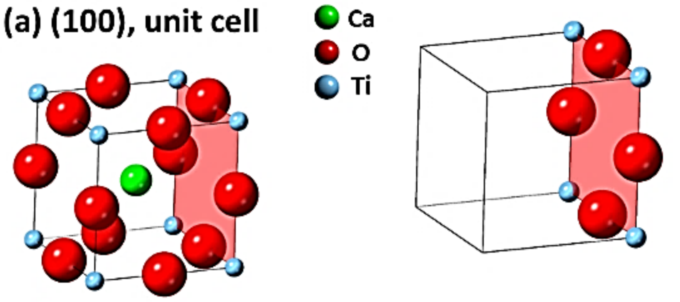

for $CaTiO_{3\,Cubic}$: (ionic radius) $r_{Ca^{2+}} = 1$ Å, $r_{Ti^{4+}} = 0.60$ Å, $r_{O^{2-}} = 1.40$ Å, $a = 3.79$ Å

area of the plane (100): $S = a \times a = 3.79 \times 3.79 = 14.36$

number of atoms in the plane (100) × area of each atom in the plane (100) $= \left[\left(4 \times \frac{1}{4} \times \pi \left(r_{O^{2-}}\right)^2\right) + \left(4 \times \frac{1}{4} \times \pi \left(r_{Ti^{4+}}\right)^2\right)\right] =$

$\left[\left((4 \times \frac{1}{4}) \times \pi\, (1.40)^2\right) + \left((4 \times \frac{1}{4}) \times \pi\, (0.60)^2\right)\right] = 13.43$

Planar density $= \dfrac{\text{number of atoms in the plane (100)} \times \text{area of each atom in the plane (100)}}{\text{area of the plane (100)}} = \dfrac{13.43}{14.36} = 0.93$

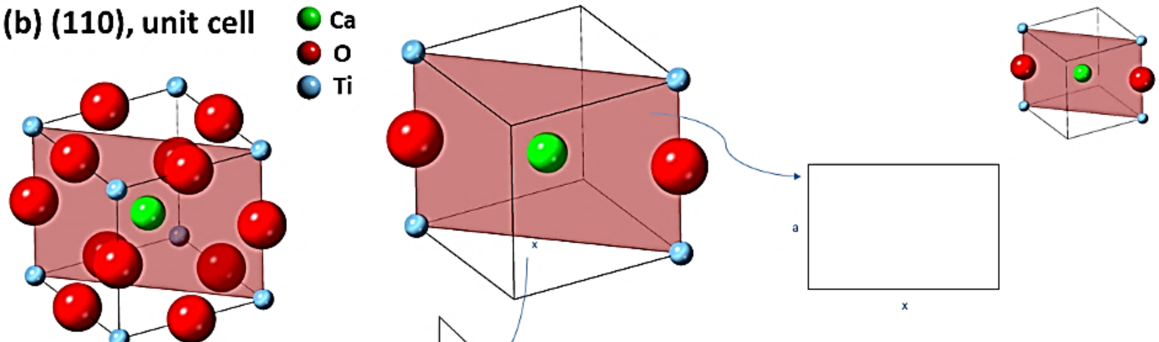

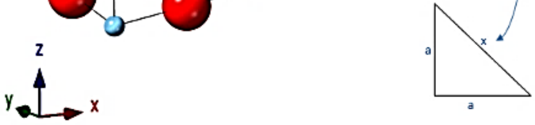

$(x)^2 = (a)^2 + (a)^2 = (3.79)^2 + (3.79)^2 \longrightarrow x = 5.35$

area of the plane (110): $S = a \times x = 3.79 \times 5.35 = 20.27$

number of atoms in the plane (110) × area of each atom in the plane (110) $= \left[\left(4 \times \frac{1}{4} \times \pi \left(r_{Ti^{4+}}\right)^2\right) + \left(2 \times \frac{1}{2} \times \pi \left(r_{O^{2-}}\right)^2\right)\right] +$

$\left[\left(1 \times \pi \left(r_{Ca^{+}}\right)^2\right)\right] = \left[\left((4 \times \frac{1}{4}) \times \pi\, (0.60)^2\right) + \left(2 \times \frac{1}{2} \times \pi\, (1.40)^2\right) + \left(1 \times \pi\, (1)^2\right)\right] = 42.10$

Planar density $= \dfrac{\text{number of atoms in the plane (110)} \times \text{area of each atom in the plane (110)}}{\text{area of the plane (110)}} = \dfrac{10.42}{20.27} = 0.51$

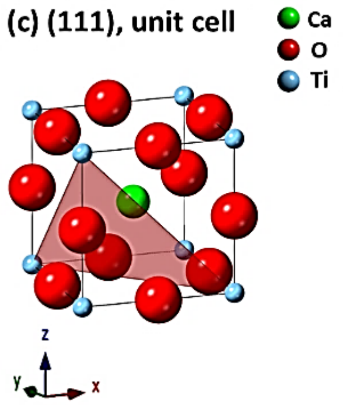

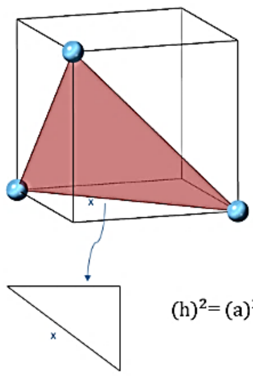

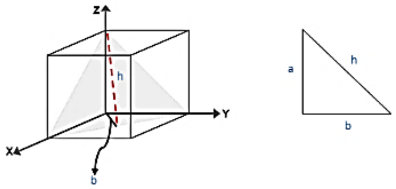

$(h)^2 = (a)^2 + (b)^2 = (3.79)^2 + (2.65)^2 \longrightarrow h = 4.62$

area of the plane (111): $S = \frac{\text{height} \times \text{width}}{2} = \frac{(h) \times x}{2} = \frac{(4.62) \times a\sqrt{2}}{2} = \frac{(4.62) \times 5.34}{2} = 12.33$

the plane (111) × area of each atom in the plane (111) $= \left[\left(3 \times \frac{1}{6}\right) \times \pi (r_{Ti^{4+}})^2\right] = 0.5 \times \pi\,(0.60)^2 = 0.56$

Planar density

planer density $= \frac{\text{...mber of atoms in the plane (111)} \times \text{area of each atom in the plane (111)}}{\text{area of the plane (111)}} = \frac{0.56}{12.33} = 0.04$

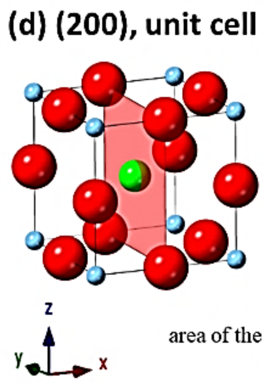

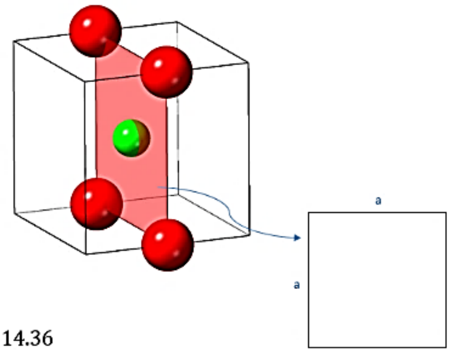

area of the plane (002): $S = a^2 = 14.36$

number of atoms in the plane (200) × area of each atom in the plane (200) $= \left[\left(4 \times \frac{1}{4} \times \pi\,(r_{O^{2-}})^2\right) + \left(1 \times \pi\,(r_{Ca^{+}})^2\right)\right] =$

[illegible] $\times \pi\,(1.40)^2\big) + (1 \times \pi\,(1)^2)\big] = 6.15 + 3.14 = 9.29$

Planar density

sity $= \frac{\text{number of atoms in the plane (002)} \times \text{area of each atom in the plane (002)}}{\text{area of the plane (002)}} = \frac{9.29}{14.36} = 0.64$

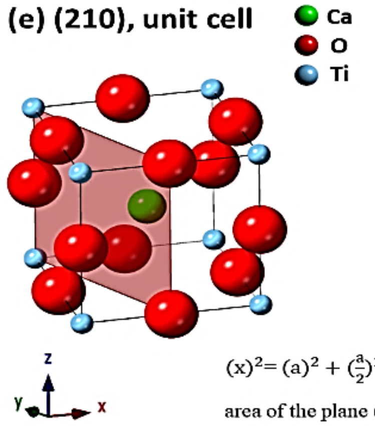

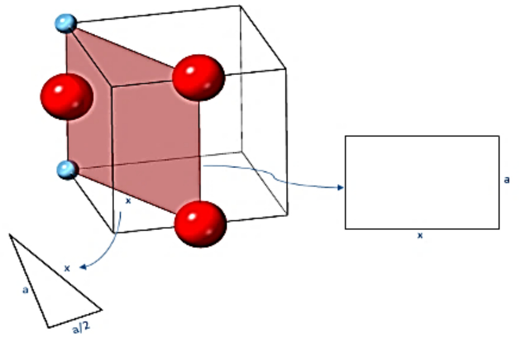

$(x)^2 = (a)^2 + (\frac{a}{2})^2 = (3.79)^2 + (1.89)^2 \longrightarrow x = 4.23$

area of the plane (210): $S = a \times x = 3.79 \times 4.23 = 16.03$

number of atoms in the plane (210) × area of each atom in the plane (210) $= \left[\left(2 \times \frac{1}{4} \times \pi\,(r_{Ti^{4+}})^2\right) + \left(1 \times \frac{1}{2} \times \pi\,(r_{O^{2-}})^2\right) + \left(2 \times \frac{1}{4} \times \pi\,(r_{O^{2-}})^2\right)\right] =$

$\left[\left(2 \times \frac{1}{4} \times \pi\,(0.60)^2\right) + \left(1 \times \frac{1}{2} \times \pi\,(1.40)^2\right) + \left(2 \times \frac{1}{4} \times \pi\,(1.40)^2\right)\right] = 6.70$

planer density $= \frac{\text{number of atoms in the plane (210)} \times \text{area of each atom in the plane (210)}}{\text{area of the plane (210)}} = \frac{6.70}{16.03} = 0.41$

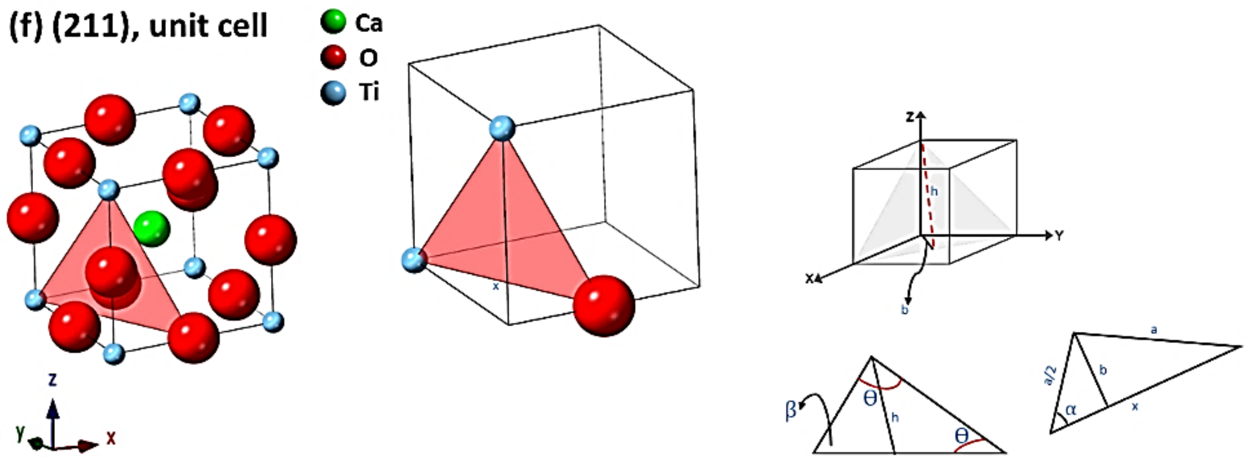

$\sin\alpha = \frac{a}{x} = \frac{3.79}{4.23} = 0.89, \quad \alpha = 62.87$ $\qquad (x)^2 = (a)^2 + (\frac{a}{2})^2 = (3.79)^2 + (1.89)^2 \longrightarrow x = 4.23$

$\sin\alpha = \frac{b}{\frac{a}{2}} = \frac{b}{1.89} = 0.89, \quad b = 1.68$

$(h)^2 = (a)^2 + (b)^2 = (3.79)^2 + (1.68)^2 \longrightarrow h = 4.14$

area of the plane (211): $S = \frac{\text{height} \times \text{width}}{2} = \frac{h \times x}{2} = \frac{(4.14) \times (4.23)}{2} = 8.75$

$\tan\beta = \frac{h}{\frac{x}{2}} = \frac{4.14}{2.11} = 1.96, \quad \beta = 62.96$ $\qquad \theta = \frac{180 - \beta}{2} = \frac{180 - 62.96}{2} = 58.52$

number of atoms in the plane (211) × area of each atom in the plane (211) $= \left[\left(1 \times \frac{\beta}{360}\right)\right] \times \pi(r_{O^{2-}})^2 + \left[\left(2 \times \frac{\theta}{360}\right)\right] \times \pi(r_{Ti^{4+}})^2 =$

$\left[\left(1 \times \frac{62.96}{360}\right)\right] \times \pi(1.40)^2 + \left[\left(2 \times \frac{58.52}{360}\right)\right] \times \pi(0.60)^2 = 1.43$

Planar density $= \frac{\text{number of atoms in the plane (211)} \times \text{area of each atom in the plane (211)}}{\text{area of the plane (211)}} = \frac{1.43}{8.75} = 0.16$

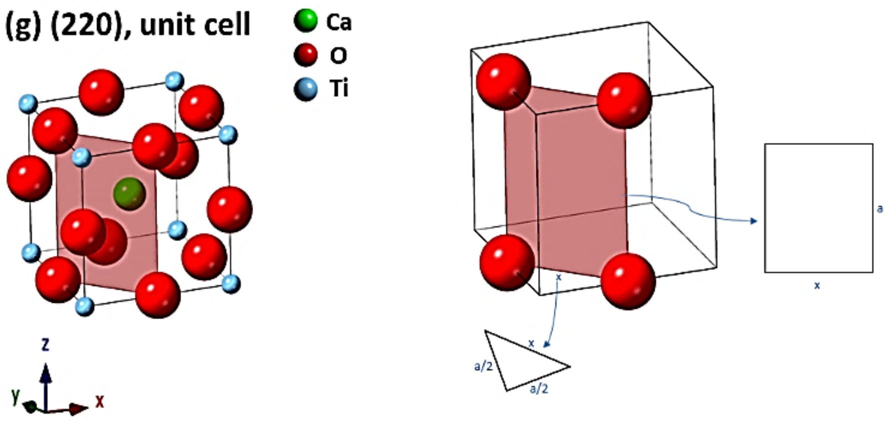

$(x)^2 = (\frac{a}{2})^2 + (\frac{a}{2})^2 = (1.89)^2 + (1.89)^2 \longrightarrow x = 2.67$

area of the plane (220): $S = a \times x = 3.79 \times 2.67 = 10.11$

number of atoms in the plane (220) × area of each atom in the plane (220) $= \left[\left(4 \times \frac{1}{4} \times \pi\left(r_{O^{2-}}\right)^2\right)\right] = \left[\left((4 \times \frac{1}{4}) \times \pi(1.40)^2\right)\right] = 6.15$

Planar density $= \frac{\text{number of atoms in the plane (220)} \times \text{area of each atom in the plane (220)}}{\text{area of the plane (220)}} = \frac{6.15}{10.11} = 0.60$

(h) (221), unit cell

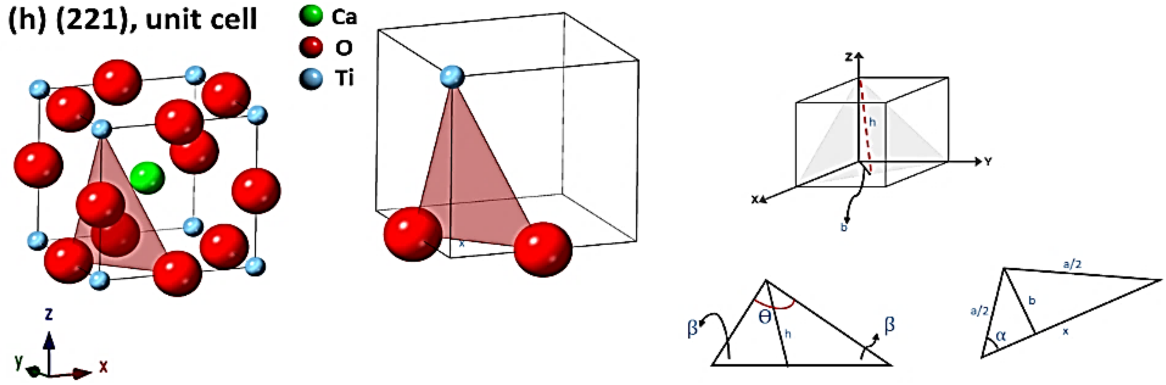

$$\sin\alpha = \frac{\frac{a}{2}}{x} = \frac{1.89}{2.67} = 0.70, \quad \alpha = 44.42 \qquad (x)^2 = (\tfrac{a}{2})^2 + (\tfrac{a}{2})^2 = (1.89)^2 + (1.89)^2, \; x = 2.67$$

$$\sin\alpha = \frac{b}{\frac{a}{2}} = \frac{b}{1.89} = 0.70, \quad b = 1.32$$

$$(h)^2 = (a)^2 + (b)^2 = (3.79)^2 + (1.32)^2, \; h = 4.01$$

$$\text{area of the plane (221): } S = \frac{\text{height} \times \text{width}}{2} = \frac{h \times x}{2} = \frac{(4.01) \times (2.67)}{2} = 5.35$$

$$\tan\beta = \frac{h}{\frac{x}{2}} = \frac{4.01}{1.33} = 3.01, \; \beta = 71.62$$

$$\theta = \frac{180 - (2\times\beta)}{2} = \frac{180 - (2\times 71.62)}{2} = 18.38$$

$$\text{number of atoms in the plane (211)} \times \text{area of each atom in the plane (211)} = \left[\left(1 \times \frac{\theta}{360}\right)\right] \times \pi(r_{Ti^{4+}})^2 + \left[\left(2 \times \frac{\beta}{360}\right)\right] \times \pi(r_{O^{2-}})^2 =$$

$$\left[\left(1 \times \frac{18.38}{360}\right)\right] \times \pi(0.60)^2 + \left[\left(2 \times \frac{71.62}{360}\right)\right] \times \pi(1.40)^2 = 2.49$$

$$\text{Planar density} = \frac{\text{number of atoms in the plane (221)} \times \text{area of each atom in the plane (221)}}{\text{area of the plane (221)}} = \frac{2.49}{5.35} = 0.46$$

(i) (310), unit cell

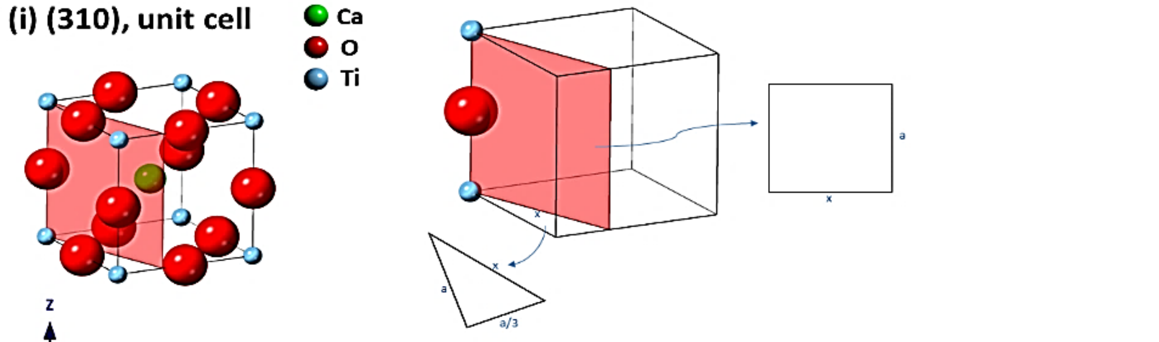

$$(x)^2 = (\tfrac{a}{3})^2 + (a)^2 = (1.26)^2 + (3.79)^2, \; x = 3.99$$

$$\text{area of the plane (310): } S = a \times x = 3.79 \times 3.99 = 15.12$$

$$\text{number of atoms in the plane (310)} \times \text{area of each atom in the plane (310)} =$$

$$\left[\left(1 \times \tfrac{1}{2} \times \pi\,(r_{O^{2-}})^2\right)\right] + \left[\left(2 \times \tfrac{1}{4} \times \pi\,(r_{Ti^{4+}})^2\right)\right] = \left[\left(2 \times \tfrac{1}{4} \times \pi\,(0.60)^2\right)\right] + \left(1 \times \tfrac{1}{2} \times \pi \times (1.4)^2\right) = 3.644$$

$$\text{Planar density} = \frac{\text{number of atoms in the plane (310)} \times \text{area of each atom in the plane (310)}}{\text{area of the plane (310)}} = \frac{3.644}{15.12} = 0.24$$

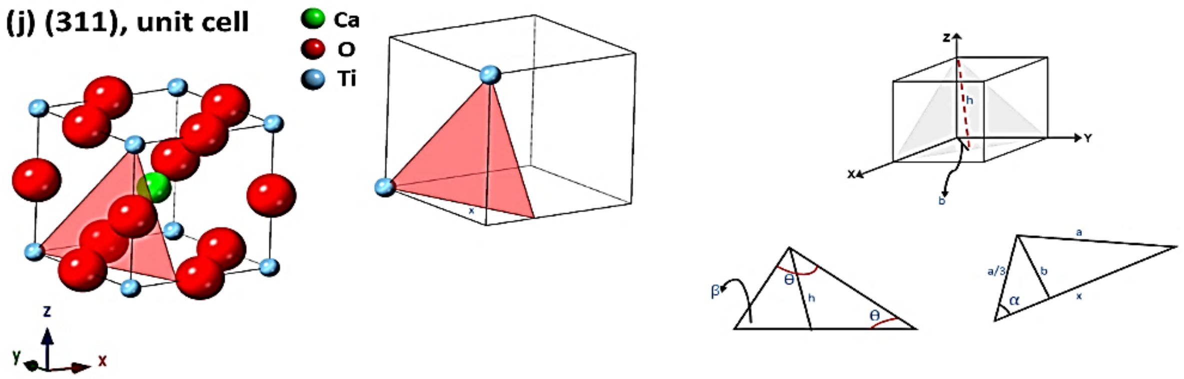

$$\sin\alpha = \frac{a}{x} = \frac{3.79}{3.99} = 0.94, \quad \alpha = 70.05 \qquad (x)^2 = \left(\frac{a}{3}\right)^2 + (a)^2 = (1.26)^2 + (3.79)^2, \; x = 3.99$$

$$\sin\alpha = \frac{b}{\frac{a}{3}} = \frac{b}{1.26} = 0.94, \quad b = 1.18$$

$$(h)^2 = (a)^2 + (b)^2 = (3.79)^2 + (1.18)^2, \; h = 3.96$$

$$\text{area of the plane (311)}: S = \frac{\text{height} \times \text{width}}{2} = \frac{h \times x}{2} = \frac{(3.96) \times (3.99)}{2} = 7.90$$

$$\tan\beta = \frac{h}{\frac{x}{2}} = \frac{3.96}{1.99} = 1.98, \; \beta = 63.20 \qquad \theta = \frac{180 - \beta}{2} = \frac{180 - (63.20)}{2} = 58.40$$

$$\text{number of atoms in the plane (311)} \times \text{area of each atom in the plane (311)} = \left[\left(2 \times \frac{\theta}{360}\right)\right] \times \pi(r_{Ti^{4+}})^2 = \left[\left(2 \times \frac{58.40}{360}\right)\right] \times \pi(0.60)^2 = 0.36$$

$$\text{Planar density} = \frac{\text{number of atoms in the plane (311)} \times \text{area of each atom in the plane (311)}}{\text{area of the plane (311)}} = \frac{0.36}{7.90} = 0.04$$

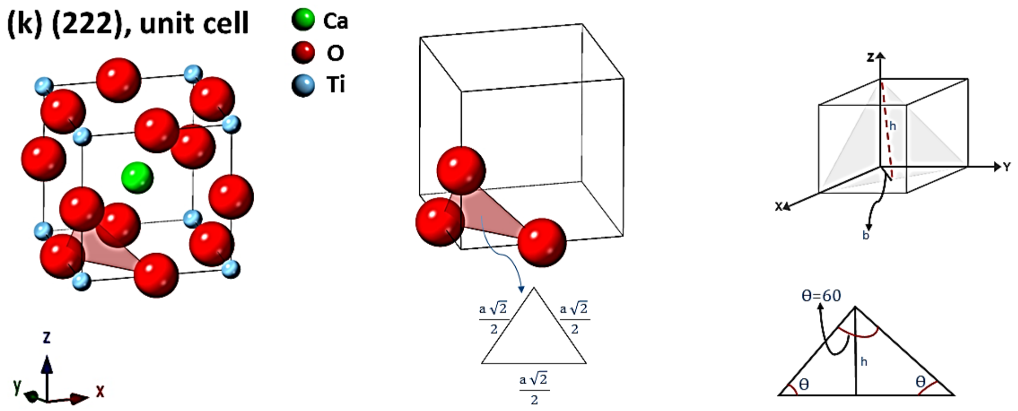

$$\left(\frac{a\sqrt{2}}{2}\right)^2 = (h)^2 + \left(\frac{a\sqrt{2}}{4}\right)^2 \longrightarrow 7.12 = (h)^2 + 1.76, \quad h = 2.31$$

$$\text{area of the plane (222)}: S = \frac{\text{height} \times \text{width}}{2} = \frac{(h) \times \frac{a\sqrt{2}}{2}}{2} = \frac{2.31 \times 2.67}{2} = 3.08$$

$$\text{number of atoms in the plane (222)} \times \text{area of each atom in the plane (222)} = \left[\left(3 \times \frac{1}{6}\right) \times \pi(r_{O^{2-}})^2\right] = \left[\left(3 \times \frac{1}{6}\right) \times \pi(1.40)^2\right] = 3.07$$

$$\text{Planar density} = \frac{\text{number of atoms in the plane (222)} \times \text{area of each atom in the plane (222)}}{\text{area of the plane (222)}} = \frac{3.07}{3.08} = 0.99$$

Figure S4. Geometry of planes and calculations of planar density of (a) (100), (b) (110), (c) (111), (d) (200), (e) (210), (f) (211), (g) (220), (h) (221), (i) (310), (j) (311) and (k) (222) related to the unit cell of $CaTiO_3$.

(a) (100), super cell (2×2×2)

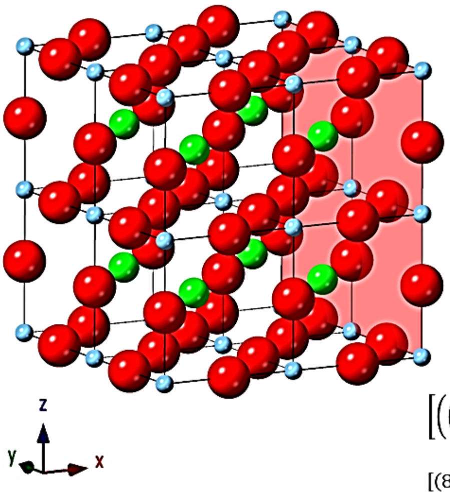

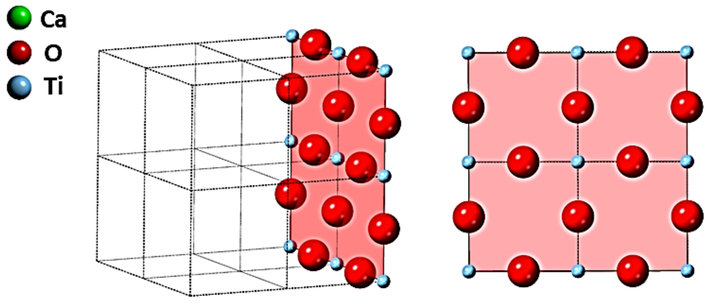

area of the plane (100): $S = 2a \times 2a = 4 \times 3.79 \times 3.79 = 57.46$

number of atoms in the plane (100) × area of each atom in the plane (100) =

$$\left[\left(\left(8\times\frac{1}{2}\right)+(4\times 1)\times\pi\left(r_{O^{2-}}\right)^2\right)+\left(\left(4\times\frac{1}{4}\right)+\left(4\times\frac{1}{2}\right)+(1)\times\pi\left(r_{Ti^{4+}}\right)^2\right)\right]=$$

$$[(8\times\pi\,(1.40)^2)+(4\times\pi\,(0.60)^2)] = 53.78$$

$$\text{Planar density} = \frac{\text{number of atoms in the plane (100)} \times \text{area of each atom in the plane (100)}}{\text{area of the plane (100)}} = \frac{53.78}{57.46} = 0.93$$

(b) (110), super cell (2×2×2)

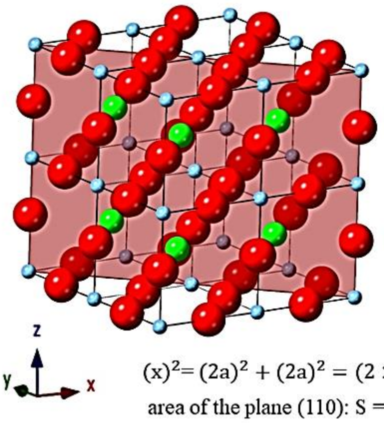

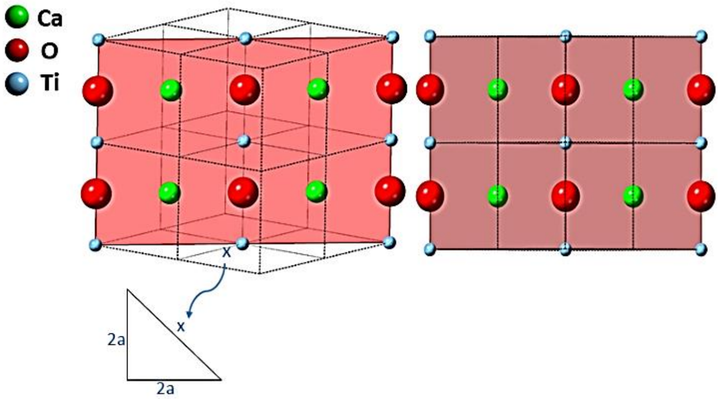

$$(x)^2 = (2a)^2 + (2a)^2 = (2\times 3.79)^2 + (2\times 3.79)^2 \longrightarrow x = 10.71$$

area of the plane (110): $S = 2a \times x = 2 \times 3.79 \times 10.71 = 81.18$

number of atoms in the plane (110) × area of each atom in the plane (110) =

$$\left[\left(\left(4\times\frac{1}{4}+4\times\frac{1}{2}+1\right)\times\pi\left(r_{Ti^{4+}}\right)^2\right)+\left(\left(4\times\frac{1}{2}+2\right)\times\pi\left(r_{O^{2-}}\right)^2\right)\right]+\left[\left(4\times\pi\times\left(r_{Ca^{+}}\right)^2\right)\right]=$$

$$[(4\times\pi\,(0.60)^2)+(4\times\pi\,(1.40)^2)+(4\times\pi\,(1)^2)] = 41.72$$

$$\text{Planar density} = \frac{\text{number of atoms in the plane (110)} \times \text{area of each atom in the plane (110)}}{\text{area of the plane (110)}} = \frac{41.72}{81.18} = 0.51$$

(c) (111), super cell (2×2×2)

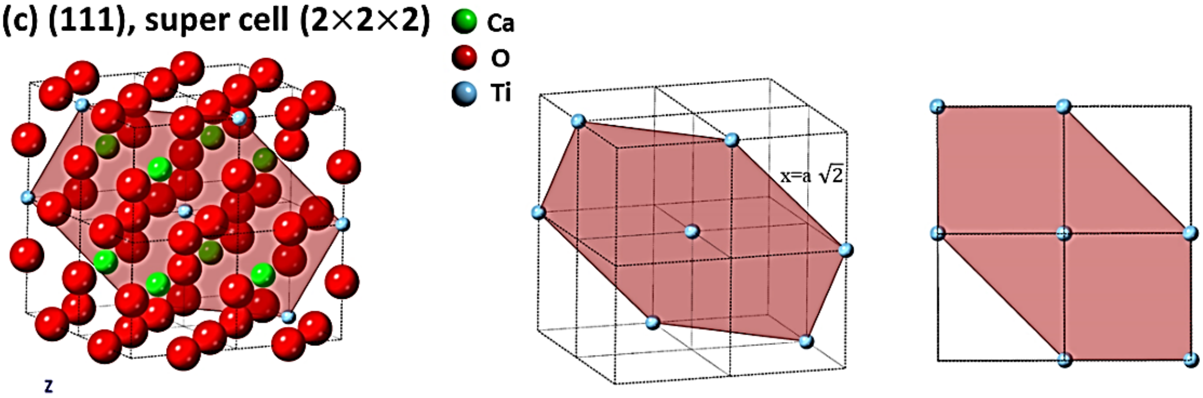

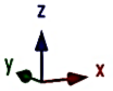

regular Hexagon area formula(each side length is equal to x)= $6 \times \frac{(x)^2\sqrt{3}}{4}$ ⟶

area of the plane (111): $S = 6 \times \frac{(3.79\times\sqrt{2})^2\times\sqrt{3}}{4} = 6 \times \frac{(28.73)\times\sqrt{3}}{4} = 76.64$

number of atoms in the plane (111) × area of each atom in the plane (111) = $\left[\left(6 \times \frac{1}{3} + 1\right) \times \pi(r_{Ti^{4+}})^2\right] = 3 \times \pi\,(0.60)^2 = 3.39$

Planar density $= \frac{\text{number of atoms in the plane (111)} \times \text{area of each atom in the plane (111)}}{\text{area of the plane (111)}} = \frac{3.39}{76.64} = 0.04$.

(d) (200), super cell (2×2×2)

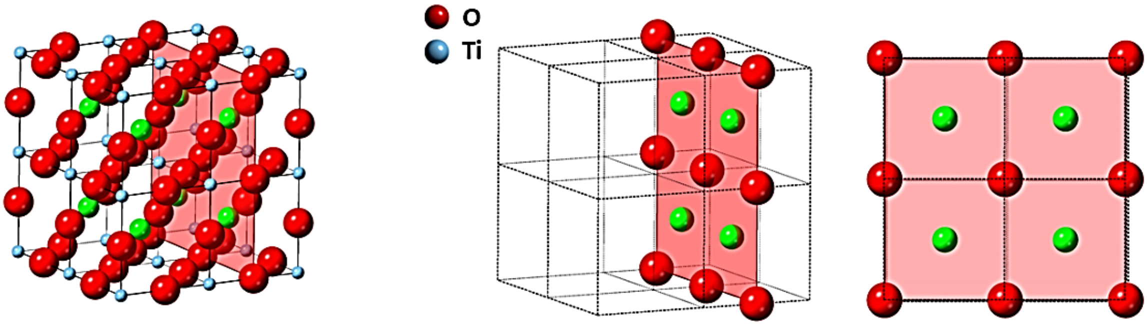

area of the plane (002): $S = (2a)^2 = 57.45$

number of atoms in the plane (200) × area of each atom in the plane (200) =

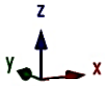

$$\left[\left((4 \times \tfrac{1}{4} + 4 \times \tfrac{1}{2} + 1) \times \pi\,(r_{O^{2-}})^2\right) + \left(4 \times \pi\,(r_{Ca^{+}})^2\right)\right] = [(4 \times \pi \times (1.40)^2) + (4 \times \pi\,(1)^2)] = 37.20$$

Planar density $= \frac{\text{number of atoms in the plane (002)} \times \text{area of each atom in the plane (002)}}{\text{area of the plane (002)}} = \frac{37.20}{57.45} = 0.64$.

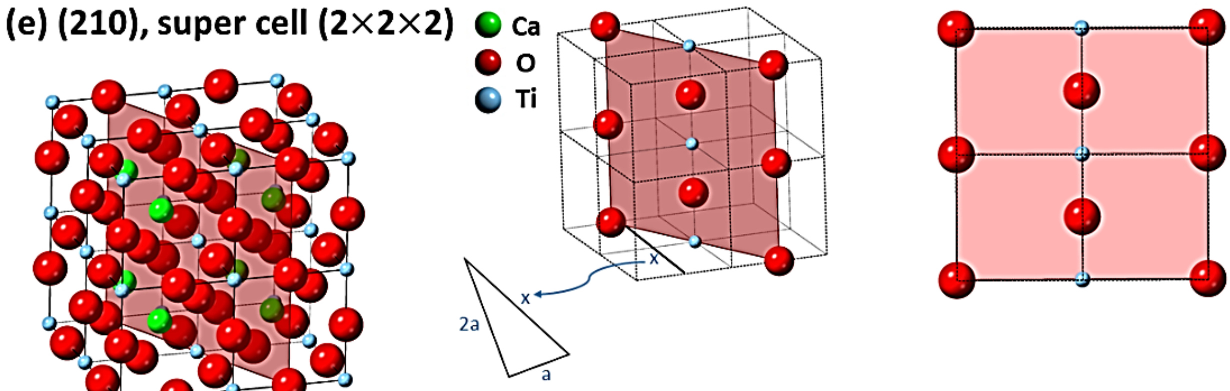

$$(x)^2 = (2a)^2 + (a)^2 = (2 \times 3.79)^2 + (3.79)^2 \longrightarrow x = 8.47$$

area of the plane (210): $S = 2a \times x = 2 \times 3.79 \times 8.47 = 64.20$

number of atoms in the plane (210) × area of each atom in the plane (210) $= \left[\left(\left(2 \times \frac{1}{2} + 1\right) \times \pi \left(r_{Ti^{4+}}\right)^2\right) + \left(\left(2 \times \frac{1}{2} + 4 \times \frac{1}{4} + 2\right) \times \pi \left(r_{O^{2-}}\right)^2\right)\right] =$
$[(2 \times \pi\, (0.60)^2) + (4 \times \pi\, (1.40)^2)] = 26.89$

Planar density $= \frac{\text{number of atoms in the plane (210)} \times \text{area of each atom in the plane (210)}}{\text{area of the plane (210)}} = \frac{26.89}{64.20} = 0.41$

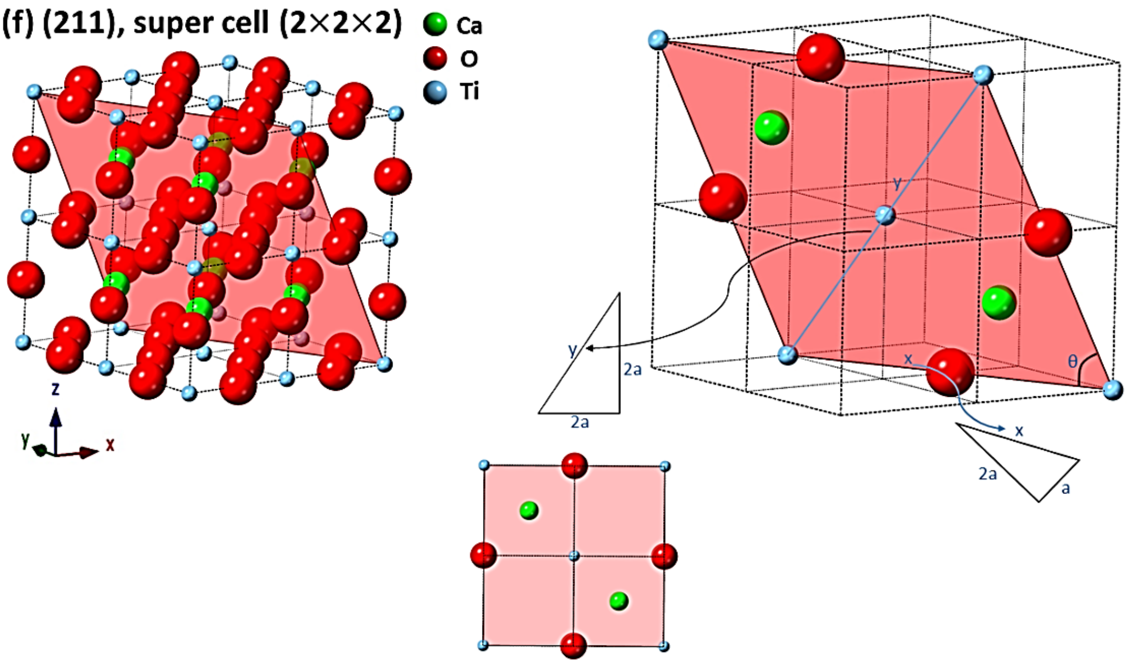

$$(x)^2 = (2a)^2 + (a)^2 = (2 \times 3.79)^2 + (3.79)^2 \longrightarrow x = 8.47$$

$$(y)^2 = (2a)^2 + (2a)^2 = (2 \times 3.79)^2 + (2 \times 3.79)^2 \longrightarrow y = 10.72$$

area of the plane (211): $s \xrightarrow{Heron's\ law} s = 2 \times \sqrt{p(p-x)(p-y)(p-z)}$

$$p = \frac{x+y+y}{2} = \frac{8.47+10.72+10.72}{2} = 14.96$$

$$s = 2 \times \sqrt{14.96\,(14.96-8.47)(14.96-10.72)(14.96-10.72)} = 2 \times 41.77 = 83.54$$

Cosines law: $y^2 = (x)^2 + (x)^2 - 2(x)(x)cos\theta \longrightarrow cos\theta = 0.199 \longrightarrow \theta = 78.52$

number of atoms in the plane (211) × area of each atom in the plane (211) =

$\left[\left(\left(2 \times \frac{78.52}{360} + 2 \times \frac{101.48}{360} + 1\right) \times \pi \left(r_{Ti^{4+}}\right)^2\right) + \left(4 \times \frac{1}{2} \times \pi \left(r_{O^{2-}}\right)^2\right)\right] + \left[\left(2 \times \pi \left(r_{Ca+}\right)^2\right)\right] = [(2 \times \pi\,(0.60)^2) + (2 \times \pi\,(1.40)^2) + (2 \times \pi\,(1)^2)] = 20.86$

Planar density $= \frac{\text{number of atoms in the plane (211)} \times \text{area of each atom in the plane (211)}}{\text{area of the plane (211)}} = \frac{20.86}{83.54} = 0.25$

(g) (220), super cell (2×2×2)

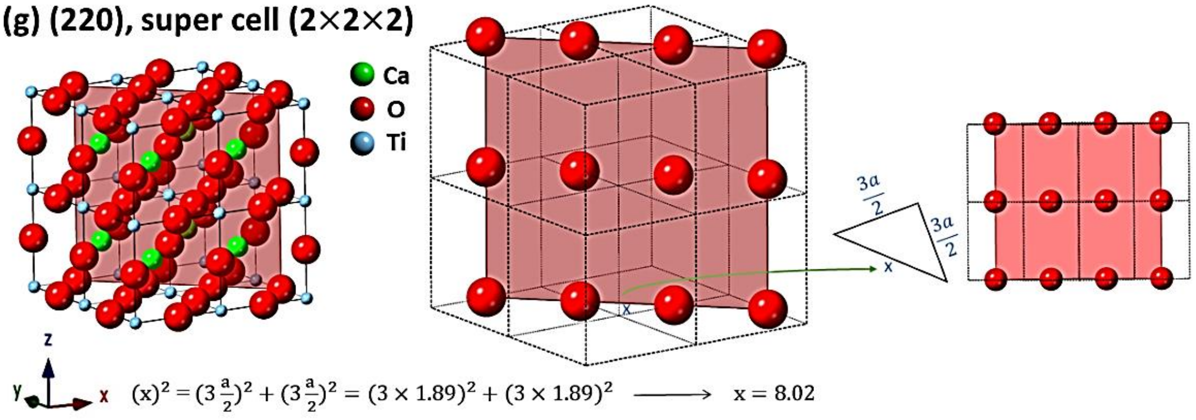

$$(x)^2 = (3\,\tfrac{a}{2})^2 + (3\,\tfrac{a}{2})^2 = (3 \times 1.89)^2 + (3 \times 1.89)^2 \longrightarrow x = 8.02$$

area of the plane (220): $S = 2a \times x = 2 \times 3.79 \times 2.67 = 60.79$

number of atoms in the plane (220) × area of each atom in the plane (220) $= \left[\left((4 \times \frac{1}{4} + 6 \times \frac{1}{2} + 2) \times \pi\, (r_{O^{2-}})^2\right)\right] =$

$[(6 \times \pi\,(1.40)^2)] = 36.94$

Planar density $= \dfrac{\text{number of atoms in the plane (220)} \times \text{area of each atom in the plane (220)}}{\text{area of the plane (220)}} = \dfrac{36.94}{60.79} = 0.60$

(h) (221), super cell (2×2×2)

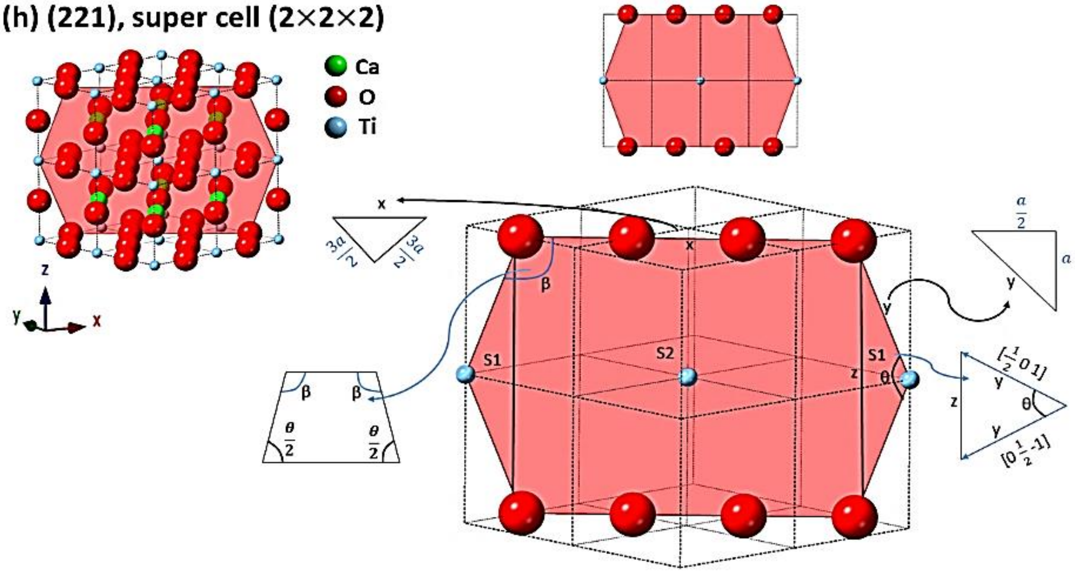

$$(x)^2 = (\tfrac{3}{2}a)^2 + (\tfrac{3}{2}a)^2 = (\tfrac{3}{2}\times 3.79)^2 + (\tfrac{3}{2}\times 3.79)^2 \longrightarrow x = 8.04$$

$$(y)^2 = (\tfrac{a}{2})^2 + (a)^2 = (1.9)^2 + (3.79)^2 \longrightarrow y = 4.24$$

area of the plane (211): $S = 2 \times S1 + S2$

Angle between two miller cubic directions: $[u_1\, v_1\, w_1] < [u_2\, v_2\, w_2]$ $\left[0\ \frac{1}{2}\ -1\right] < \left[-\frac{1}{2}\ 0\ 1\right]$

$$cos\theta = \frac{[u_1u_2 + v_1v_2 + w_1w_2]}{\sqrt{(u_1^2+v_1^2+w_1^2)}\sqrt{(u_2^2+v_2^2+w_2^2)}} = \frac{-1}{\sqrt{\left(\frac{1}{4}+1\right)\times(\frac{1}{4}+1)}} = -0.8 \longrightarrow \theta = 143.13 \longrightarrow \beta = \left(\frac{360-(143.13)}{2}\right) = 108.44$$

$$S1 = \frac{1}{2}\times y\times y\times \sin\theta = \frac{1}{2}\times 4.24\times 4.24\times \sin(143.13) = 5.39$$

Cosines law: $z^2 = (y)^2 + (y)^2 - 2(y)(y)cos\theta \longrightarrow z = 8.04 \longrightarrow S2 = z\times x = 8.04\times 8.04 = 64.64 \longrightarrow S = 64.64 + 2\times 5.39 = 75.42$

number of atoms in the plane (211) × area of each atom in the plane (211) =

$$\left[\left(\left(2\times\frac{143.13}{360}+1\right)\times\pi\,(r_{Ti^{4+}})^2\right)+\left(\left(4\times\frac{108.44}{360}+4\times\frac{1}{2}\right)\times\pi\,(r_{O^{2-}})^2\right)\right] = [(1.8\times\pi\,(0.60)^2)+(3.2\times\pi\,(1.40)^2)] = 21.74$$

Planar density $= \frac{\text{number of atoms in the plane (211)} \times \text{area of each atom in the plane (211)}}{\text{area of the plane (211)}} = \frac{21.74}{75.42} = 0.29$

(i) (310), super cell (2×2×2)

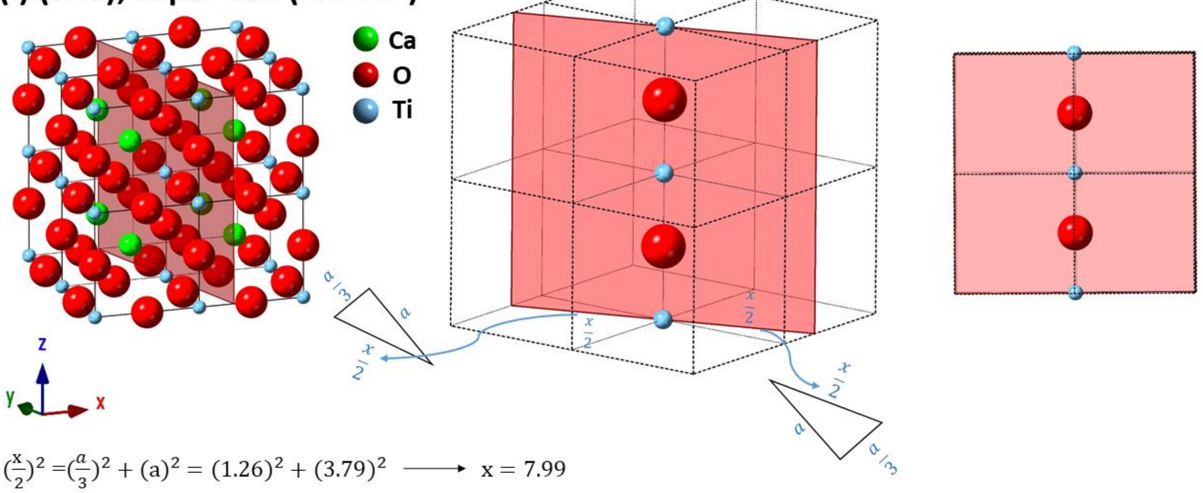

$$(\tfrac{x}{2})^2 = (\tfrac{a}{3})^2 + (a)^2 = (1.26)^2 + (3.79)^2 \longrightarrow x = 7.99$$

area of the plane (310): $S = 2a\times x = 2\times 3.79\times 7.99 = 60.56$

number of atoms in the plane (310) × area of each atom in the plane (310) $= \left[\left(2\times\pi\,(r_{O^{2-}})^2\right)\right] + \left[\left((2\times\frac{1}{2}+1)\times\pi\,(r_{Ti^{4+}})^2\right)\right]$

$= [(2\times\pi\,(1.4)^2)] + (2\times\pi\times(0.6)^2) = 14.58$

Planar density $= \frac{\text{number of atoms in the plane (310)} \times \text{area of each atom in the plane (310)}}{\text{area of the plane (310)}} = \frac{14.58}{60.56} = 0.24$

(j) (311), super cell (2×2×2)

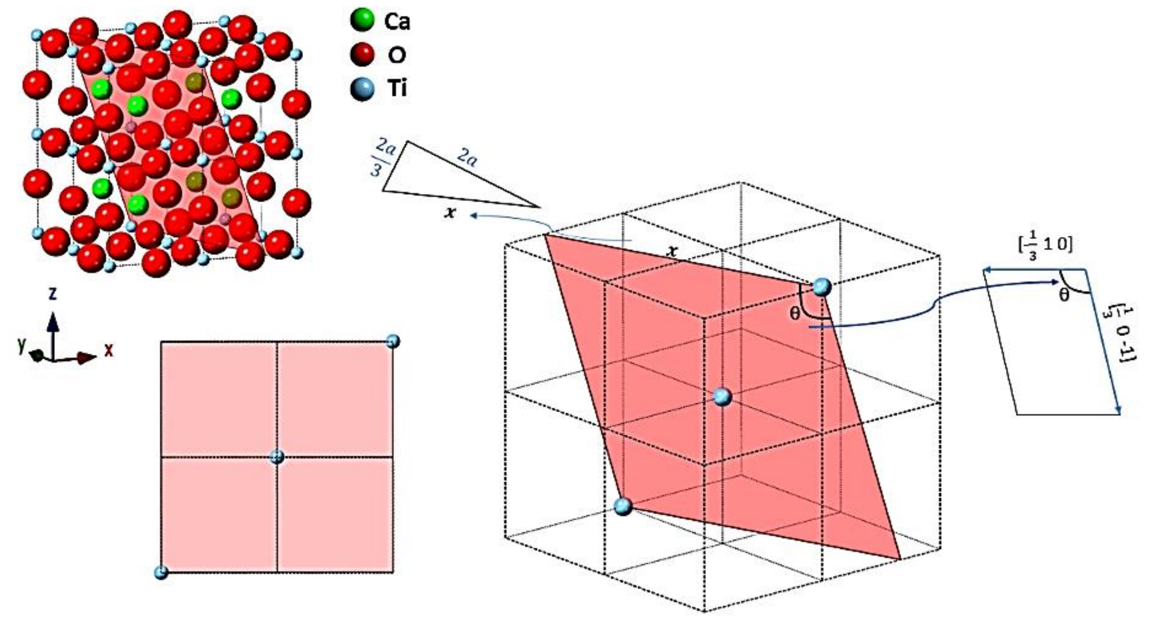

$(x)^2 = (\frac{2a}{3})^2 + (2a)^2 = (2.53)^2 + (7.58)^2 \longrightarrow x = 7.99$

Angle between two miller cubic directions: $[u_1\, v_1\, w_1] < [u_2\, v_2\, w_2]$ $\left[-\frac{1}{3}\ 1\ 0\right] \measuredangle \left[\frac{1}{3}\ 0\ -1\right]$

$$cos\theta = \frac{[u_1u_2 + v_1v_2 + w_1w_2]}{\sqrt{(u_1^2+v_1^2+w_1^2)}\sqrt{(u_2^2+v_2^2+w_2^2)}} = \frac{-\frac{1}{9}}{\sqrt{\left(\frac{1}{9}+1\right)\times\left(\frac{1}{9}+1\right)}} = -0.1 \longrightarrow \theta = 95.74$$

area of the plane (311): $S = x \times x \times \sin\theta = 7.99 \times 7.99 \times \sin 95.74 = 63.52$

number of atoms in the plane (311) × area of each atom in the plane (311) $= \left[\left((2\times\frac{95.74}{360}+1)\times\pi\left(r_{Ti^{4+}}\right)^2\right)\right] = (2\times\pi\times(0.6)^2) = 1.73$

Planar density $= \frac{\text{number of atoms in the plane (311)} \times \text{area of each atom in the plane (311)}}{\text{area of the plane (311)}} = \frac{1.73}{63.52} = 0.03$

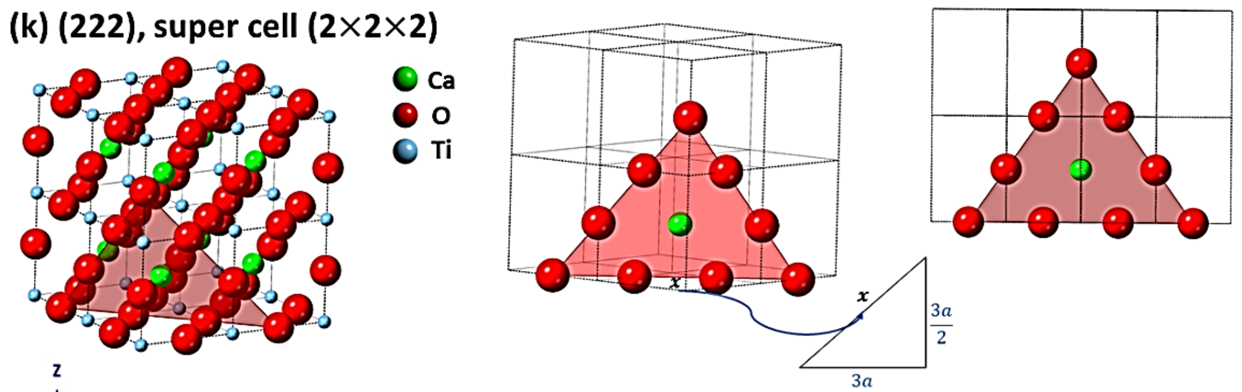

y x $(x)^2 = (3\frac{a}{2})^2 + (3\frac{a}{2})^2 = (3 \times 1.89)^2 + (3 \times 1.89)^2 \longrightarrow x = 8.02$

area of the plane (222): $S = 0.5 \times x \times x \times \sin 60 = 0.5 \times 8.02 \times 8.02 \times 0.866 = 27.85$

number of atoms in the plane (222) × area of each atom in the plane (220) = $\left[\left((3 \times \frac{1}{6} + 6 \times \frac{1}{2}) \times \pi\,(r_{O^{2-}})^2\right)\right] + \left[\left(1 \times \pi \times (r_{Ca^{+}})^2\right)\right] =$

$[(3.5 \times \pi\,(1.40)^2)] + [(1 \times \pi\,(1)^2)] = 24.69$

planer density $= \frac{\text{number of atoms in the plane (222)} \times \text{area of each atom in the plane (222)}}{\text{area of the plane (222)}} = \frac{24.69}{27.85} = 0.88$

Figure S5. Geometry of planes and calculations of planar density of (a) (100), (b) (110), (c) (111), (d) (200), (e) (210), (f) (211), (g) (220), (h) (221), (i) (310), (j) (311) and (k) (222) related to the super cells (2×2×2) of $CaTiO_3$.

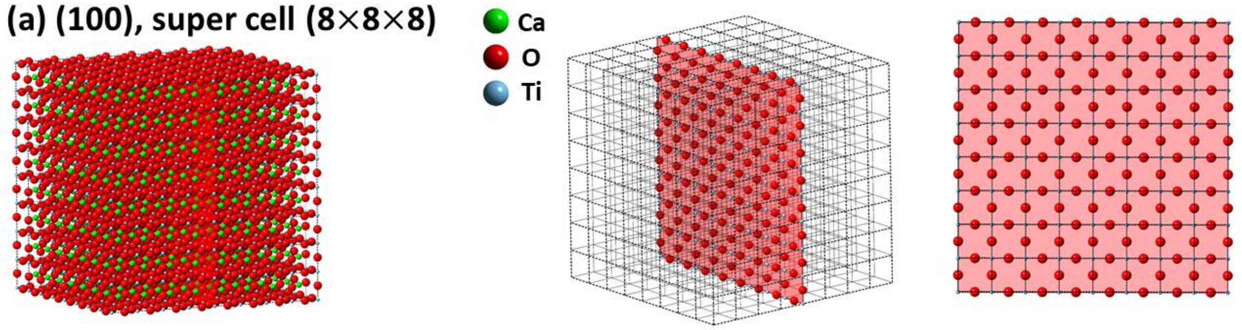

z
y x

area of the plane (100): $S = 8a \times 8a = 64 \times 3.79 \times 3.79 = 919.30$

number of atoms in the plane (100) × area of each atom in the plane (100) =

$\left[\left(\left(32 \times \frac{1}{2}\right) + (112 \times 1) \times \pi\,(r_{O^{2-}})^2\right) + \left(\left(4 \times \frac{1}{4}\right) + \left(28 \times \frac{1}{2}\right) + (49) \times \pi\,(r_{Ti^{4+}})^2\right)\right] =$

$[(128 \times \pi\,(1.40)^2) + (64 \times \pi\,(0.60)^2)] = 860.54$

Planar density $= \frac{\text{number of atoms in the plane (100)} \times \text{area of each atom in the plane (100)}}{\text{area of the plane (100)}} = \frac{860.54}{919.30} = 0.93$

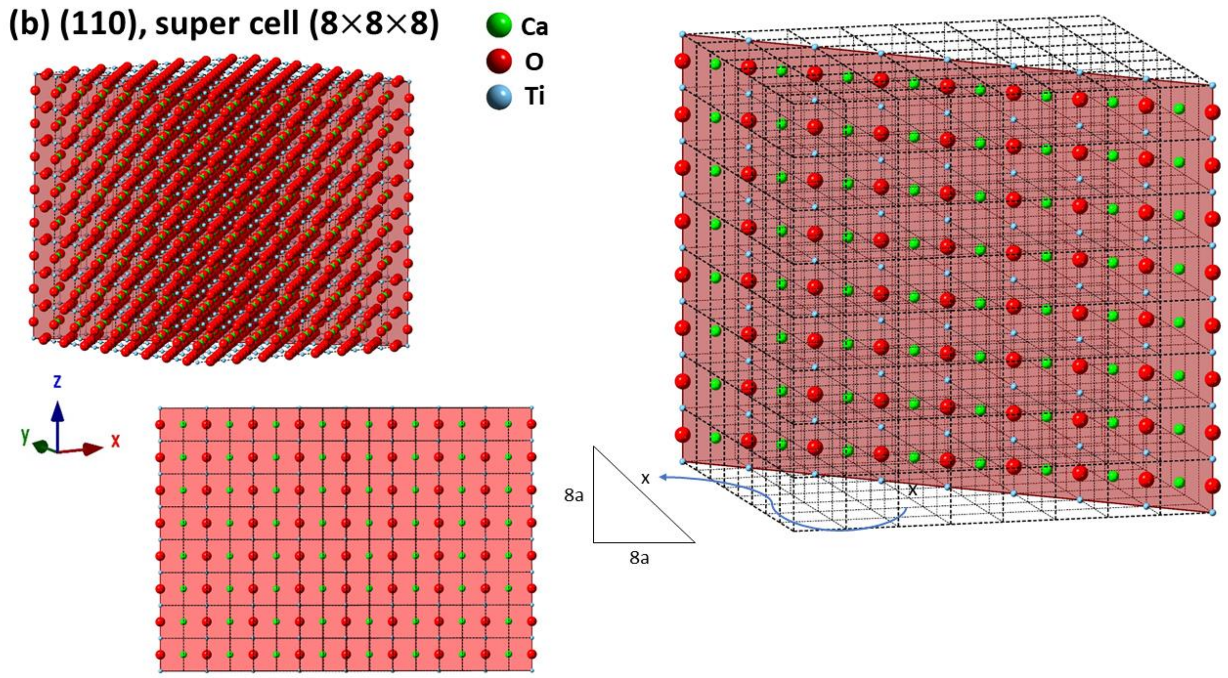

$$(x)^2 = (8a)^2 + (8a)^2 = (8 \times 3.79)^2 + (8 \times 3.79)^2 \longrightarrow x = 42.88$$

area of the plane (110): $S = 8a \times x = 8 \times 3.79 \times 42.88 = 1300.12$

number of atoms in the plane (110) × area of each atom in the plane (110) =

$$\left[\left(\left(4 \times \frac{1}{4} + 28 \times \frac{1}{2} + 49\right) \times \pi \left(r_{Ti^{4+}}\right)^2\right) + \left(\left(16 \times \frac{1}{2} + 56\right) \times \pi \left(r_{O^{2-}}\right)^2\right)\right] + \left[\left(64 \times \pi \times \left(r_{Ca^{+}}\right)^2\right)\right] =$$

$$[(64 \times \pi\ (0.60)^2) + (64 \times \pi\ (1.40)^2) + (64 \times \pi\ (1)^2)] = 667.52$$

Planar density $= \dfrac{\text{number of atoms in the plane (110)} \times \text{area of each atom in the plane (110)}}{\text{area of the plane (110)}} = \dfrac{667.52}{1300.12} = 0.51$

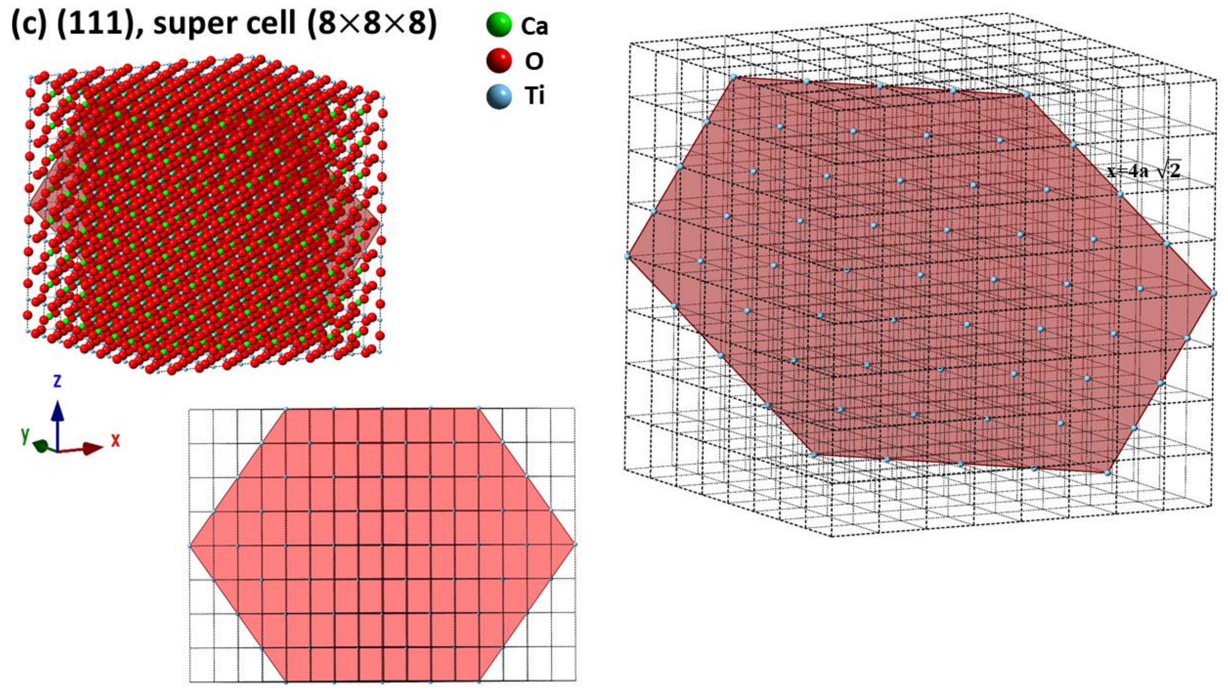

regular Hexagon area formula(each side length is equal to x)= $6 \times \frac{(x)^2\sqrt{3}}{4}$ ⟶

area of the plane (111): $S = 6 \times \frac{(4\times3.79\times\sqrt{2})^2\times\sqrt{3}}{4} = 6 \times \frac{(459.65)\times\sqrt{3}}{4} = 1194.21$

number of atoms in the plane (111) × area of each atom in the plane (111) = $\left[\left(6 \times \frac{1}{3} + 18 \times \frac{1}{2} + 37\right) \times \pi(\, r_{Ti^{4+}}\,)^2\right] =$

$48 \times \pi\,(0.60)^2 = 54.28$

Planar density $= \frac{\text{number of atoms in the plane (111)} \times \text{area of each atom in the plane (111)}}{\text{area of the plane (111)}} = \frac{54.28}{1194.21} = 0.04$

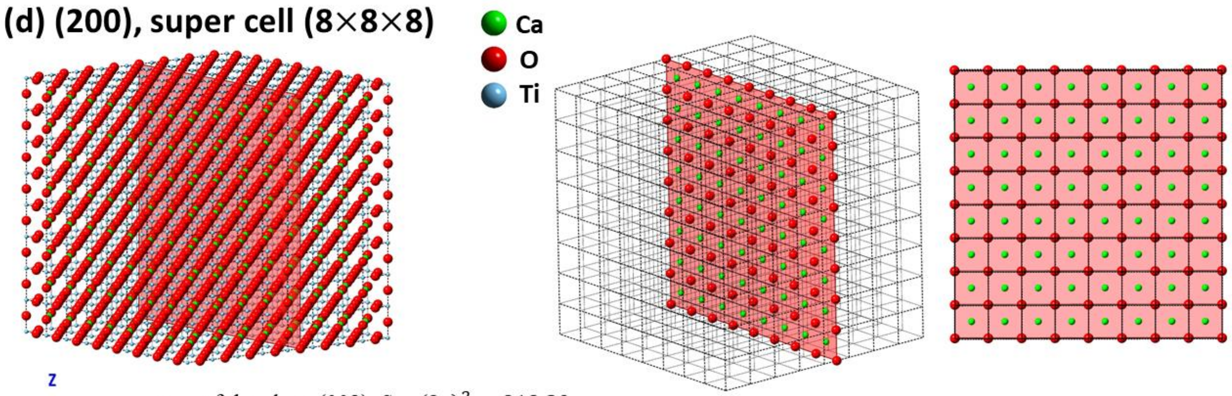

area of the plane (002): $S = (8a)^2 = 919.30$

number of atoms in the plane (200) × area of each atom in the plane (200) =

$\left[\left((4 \times \frac{1}{4} + 28 \times \frac{1}{2} + 49) \times \pi\,\left(r_{O^{2-}}\right)^2\right) + \left(4 \times \pi\,\left(r_{Ca^{+}}\right)^2\right)\right] = [(64 \times \pi \times (1.40)^2) + (64 \times \pi\,(1)^2)] = 595.14$

Planar density $= \frac{\text{number of atoms in the plane (002)} \times \text{area of each atom in the plane (002)}}{\text{area of the plane (002)}} = \frac{595.14}{919.30} = 0.64$

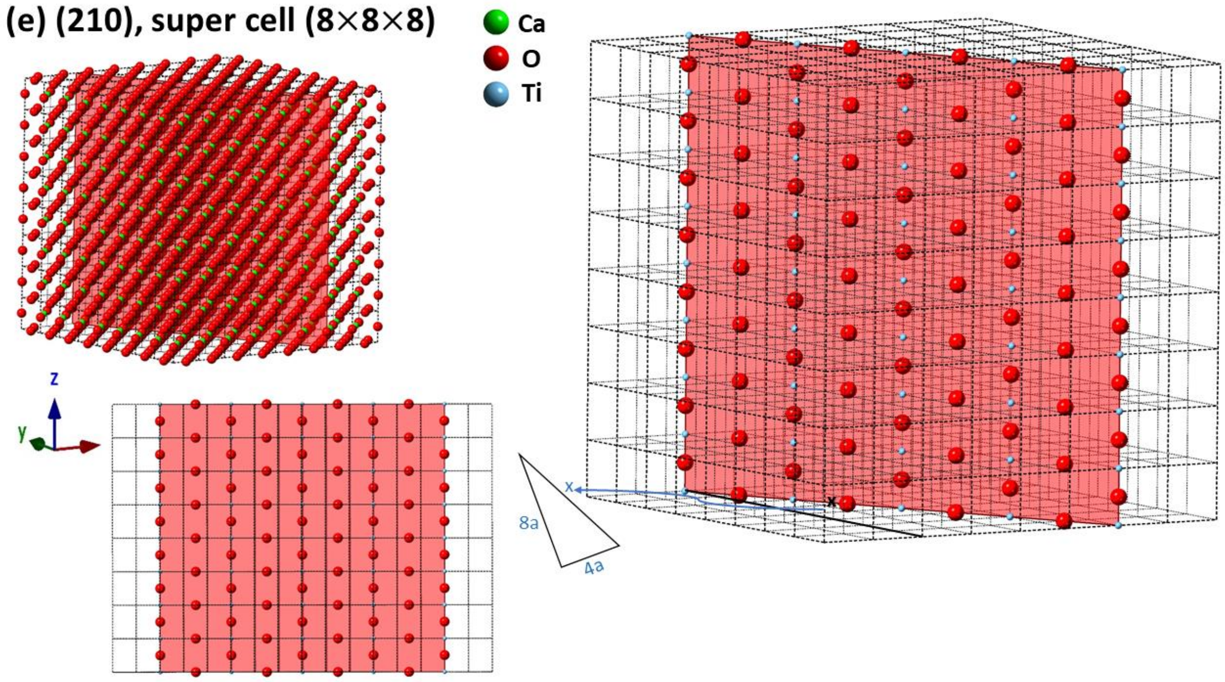

.

$(x)^2 = (8a)^2 + (4a)^2 = (8 \times 3.79)^2 + (4 \times 3.79)^2 \longrightarrow x = 33.90$

area of the plane (210): $S = 8a \times x = 8 \times 3.79 \times 33.90 = 1027.85$

number of atoms in the plane (210) × area of each atom in the plane (210) =

$\left[\left((20 \times \frac{1}{2} + 4 \times \frac{1}{4} + 21) \times \pi\left(r_{Ti^{4+}}\right)^2\right) + \left((24 \times \frac{1}{2} + 52) \times \pi\left(r_{O^{2-}}\right)^2\right)\right] = [(32 \times \pi\ (0.60)^2) + (64 \times \pi\ (1.40)^2)] = 430.27$

$\text{planer density} = \frac{\text{number of atoms in the plane (210)} \times \text{area of each atom in the plane (210)}}{\text{area of the plane (210)}} = \frac{430.27}{1027.85} = 0.41$

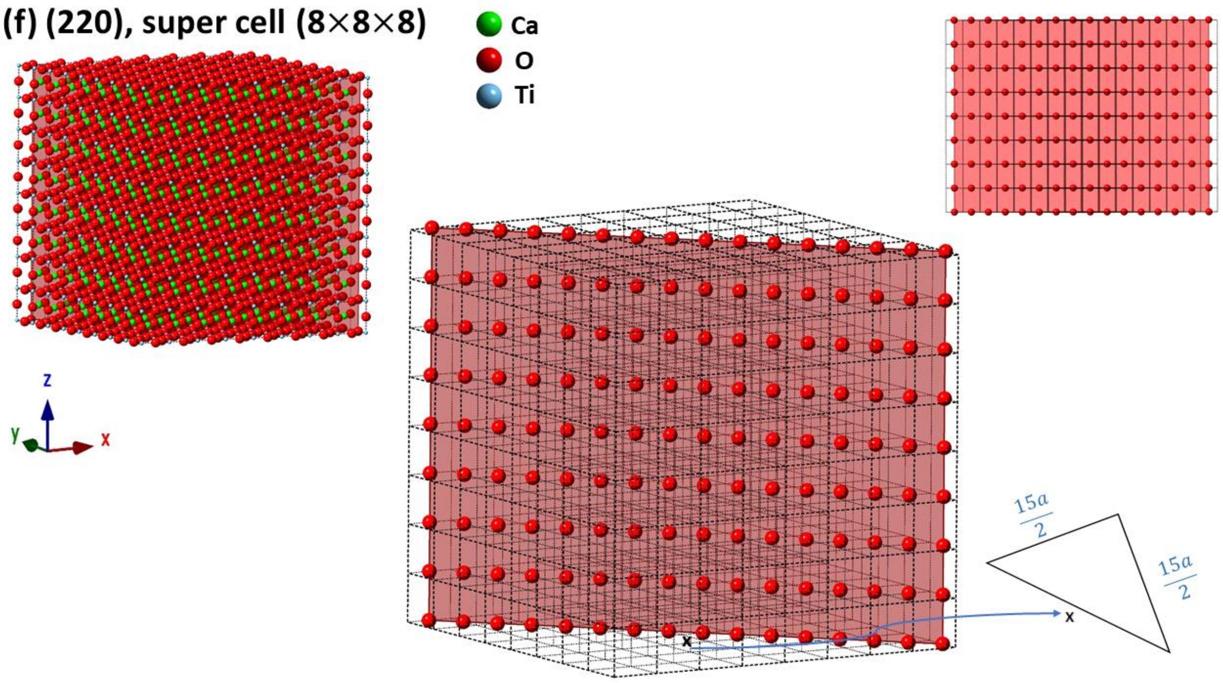

$(x)^2 = (15\frac{a}{2})^2 + (15\frac{a}{2})^2 = (15 \times 1.89)^2 + (15 \times 1.89)^2 \longrightarrow x = 40.09$

area of the plane (220): $S = 8a \times x = 8 \times 3.79 \times 40.09 = 1215.53$

number of atoms in the plane (220) × area of each atom in the plane (220) = $\left[\left((4 \times \frac{1}{4} + 42 \times \frac{1}{2} + 98) \times \pi\left(r_{O^{2-}}\right)^2\right)\right] =$ $[(120 \times \pi\ (1.40)^2)] = 738.90$

$\text{Planar density} = \frac{\text{number of atoms in the plane (220)} \times \text{area of each atom in the plane (220)}}{\text{area of the plane (220)}} = \frac{738.90}{1215.53} = 0.60$

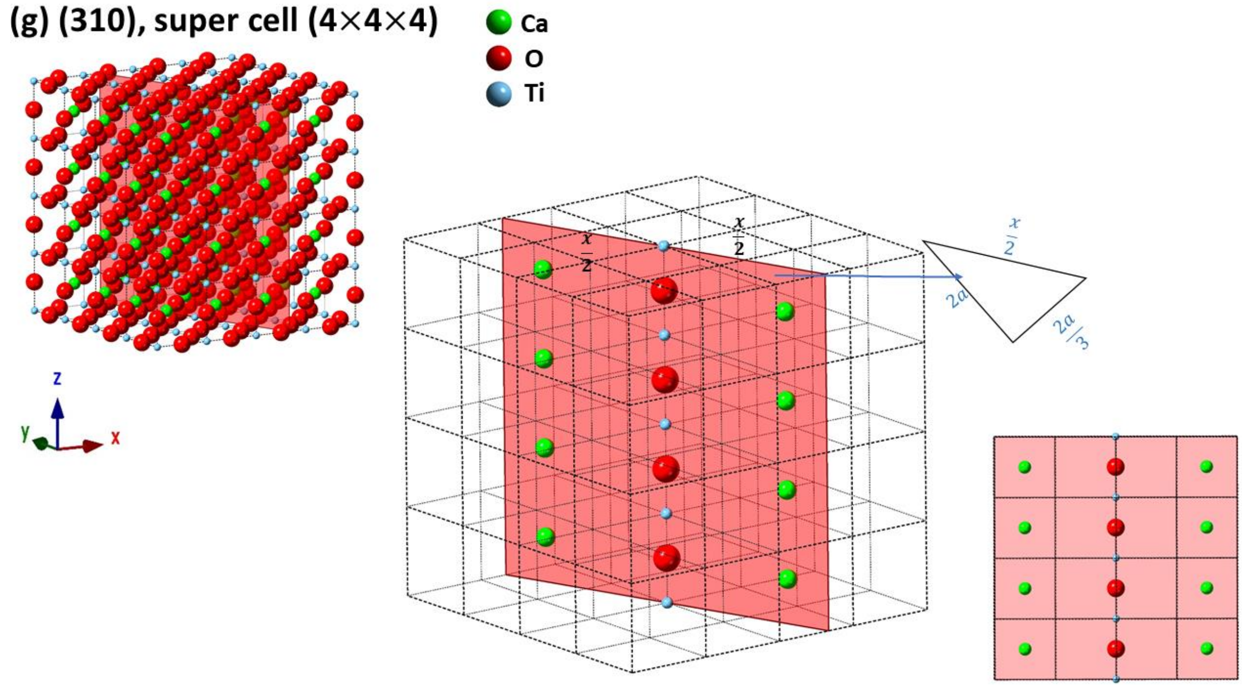

$$(\frac{x}{2})^2 = (2\frac{a}{3})^2 + (2a)^2 = (2.52)^2 + (7.58)^2 \longrightarrow x = 15.97$$

area of the plane (310): $S = 4a \times x = 4 \times 3.79 \times 15.97 = 242.10$

number of atoms in the plane (310) × area of each atom in the plane (310) $= \left[\left(4 \times \pi \left(r_{O^{2-}}\right)^2\right)\right] + \left[\left((2 \times \frac{1}{2} + 3) \times \pi \left(r_{Ti^{4+}}\right)^2\right)\right]$

$\left[\left(8 \times \pi \left(r_{Ca+}\right)^2\right)\right] = [(4 \times \pi\ (1.4)^2)] + (4 \times \pi \times (0.6)^2) + (8 \times \pi \times (1)^2) = 54.30$

Planar density $= \frac{\text{number of atoms in the plane (310)} \times \text{area of each atom in the plane (310)}}{\text{area of the plane (310)}} = \frac{54.30}{242.10} = 0.23$

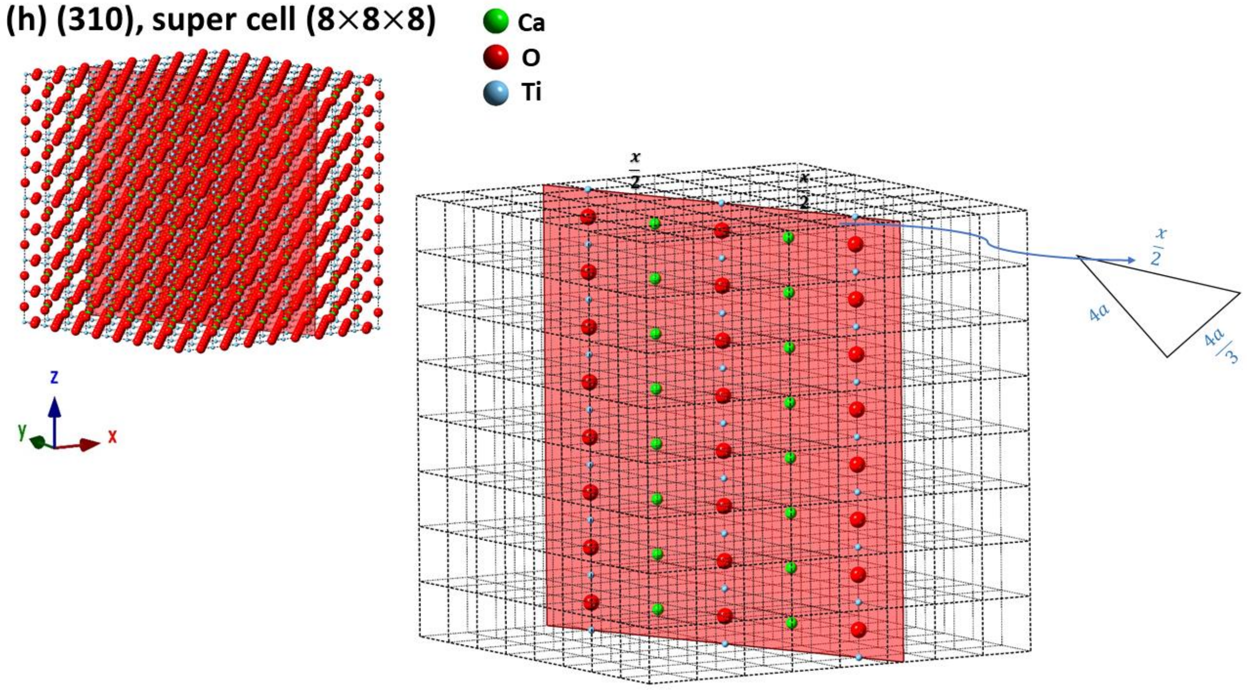

.

$(\frac{x}{2})^2 = (4\frac{a}{3})^2 + (4a)^2 = (5.05)^2 + (15.16)^2 \longrightarrow x = 31.96$

area of the plane (310): $S = 8a \times x = 8 \times 3.79 \times 31.96 = 969.03$

number of atoms in the plane (310) × area of each atom in the plane (310) = $\left[\left(24 \times \pi \left(r_{O^{2-}}\right)^2\right)\right] + \left[\left((6 \times \frac{1}{2} + 21) \times \pi \left(r_{Ti^{4+}}\right)^2\right)\right]$

$\left[\left(16 \times \pi \left(r_{Ca+}\right)^2\right)\right] = [(24 \times \pi\,(1.4)^2)] + (24 \times \pi \times (0.6)^2) + (16 \times \pi \times (1)^2) = 225.18$

Planar density $= \frac{\text{number of atoms in the plane (310)} \times \text{area of each atom in the plane (310)}}{\text{area of the plane (310)}} = \frac{225.18}{969.03} = 0.23$

.

Figure S6. Geometry of planes and calculations of planar density of (a) (100), (b) (110), (c) (111), (d) (200), (e) (210), (f) (220), (g) (310) (4×4×4) and (h) (310) (8×8×8) related to the super cells (8×8×8) of $CaTiO_3$.

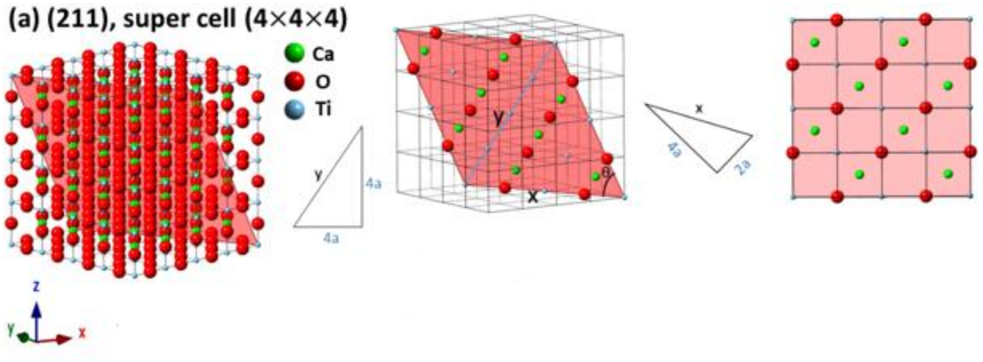

$(x)^2 = (2a)^2 + (4a)^2 = (2 \times 3.79)^2 + (4 \times 3.79)^2 \longrightarrow x = 16.95$

$(y)^2 = (4a)^2 + (4a)^2 = (4 \times 3.79)^2 + (4 \times 3.79)^2 \longrightarrow y = 21.44$

area of the plane (211): $s \xrightarrow{Heron's\ law} s = 2 \times \sqrt{p(p-x)(p-y)(p-z)}$

$p = \frac{x+y+y}{2} = \frac{16.95+21.44+21.44}{2} = 29.92$

$s = 2 \times \sqrt{29.92(29.92-16.95)(29.92-21.44)(29.92-21.44)} = 2 \times 167.05 = 334.1$

Cosines law: $y^2 = (x)^2 + (x)^2 - 2(x)(x)cos\theta \longrightarrow cos\theta = 0.199 \longrightarrow \theta = 78.52$

number of atoms in the plane (211) × area of each atom in the plane (211) =

$\left[\left((2 \times \frac{78.52}{360} + 2 \times \frac{101.48}{360} + 4 \times \frac{1}{2} + 5) \times \pi \left(r_{Ti^{4+}}\right)^2\right) + \left((8 \times \frac{1}{2} + 4) \times \pi \left(r_{O^{2-}}\right)^2\right)\right] + \left[\left(8 \times \pi \left(r_{Ca+}\right)^2\right)\right] =$

$[(8 \times \pi\,(0.60)^2) + (8 \times \pi\,(1.40)^2) + (8 \times \pi\,(1)^2)] = 83.44$

Planar density $= \frac{\text{number of atoms in the plane (211)} \times \text{area of each atom in the plane (211)}}{\text{area of the plane (211)}} = \frac{83.44}{334.1} = 0.25$

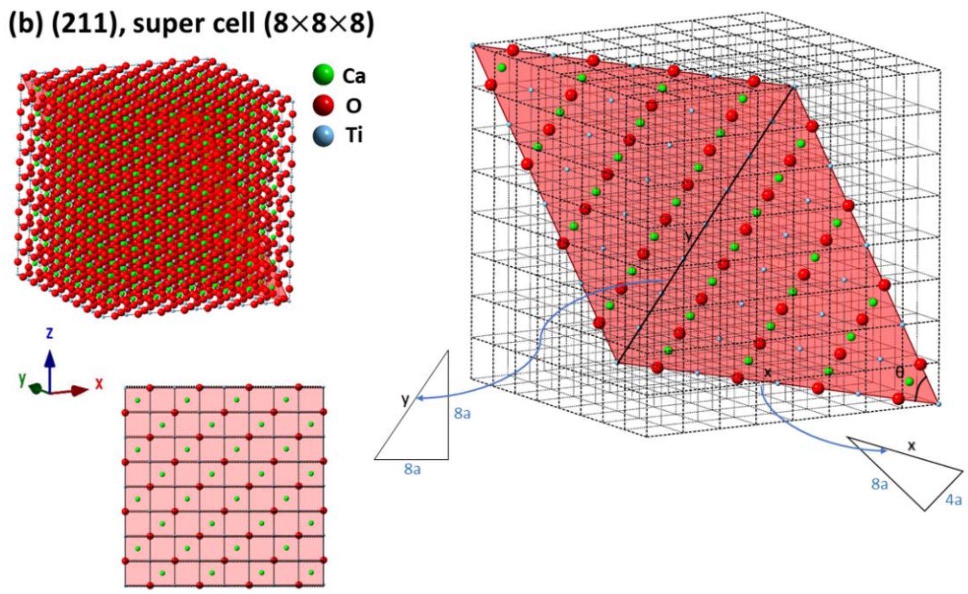

$$(x)^2 = (4a)^2 + (8a)^2 = (4 \times 3.79)^2 + (8 \times 3.79)^2 \longrightarrow x = 33.90$$

$$(y)^2 = (8a)^2 + (8a)^2 = (8 \times 3.79)^2 + (8 \times 3.79)^2 \longrightarrow y = 42.88$$

area of the plane (211): $s \xrightarrow{Heron's\ law} s = 2 \times \sqrt{p(p-x)(p-y)(p-z)}$

$$p = \frac{x+y+y}{2} = \frac{33.90+42.88+42.88}{2} = 59.83$$

$$s = 2 \times \sqrt{59.83(59.83-33.90)(59.83-42.88)(59.83-42.88)} = 2 \times 667.62 = 1335.24$$

Cosines law: $y^2 = (x)^2 + (x)^2 - 2(x)(x)cos\theta \longrightarrow cos\theta = 0.199 \longrightarrow \theta = 78.52$

number of atoms in the plane (211) × area of each atom in the plane (211) =

$$\left[\left((2 \times \tfrac{78.52}{360} + 2 \times \tfrac{101.48}{360} + 12 \times \tfrac{1}{2} + 25) \times \pi\, (r_{Ti^{4+}})^2\right) + \left((16 \times \tfrac{1}{2} + 24) \times \pi\, (r_{O^{2-}})^2\right)\right] + \left[\left(32 \times \pi\, (r_{Ca^{+}})^2\right)\right] =$$

$$[(32 \times \pi\, (0.60)^2) + (32 \times \pi\, (1.40)^2) + (32 \times \pi\, (1)^2)] = 333.76$$

$$\text{Planar density} = \frac{\text{number of atoms in the plane (211)} \times \text{area of each atom in the plane (211)}}{\text{area of the plane (211)}} = \frac{333.76}{1335.24} = 0.25$$

Figure S7. Geometry of planes and calculations of planar density of (a) (211) super cell (4×4×4) and (b) (211) super cell (8×8×8).

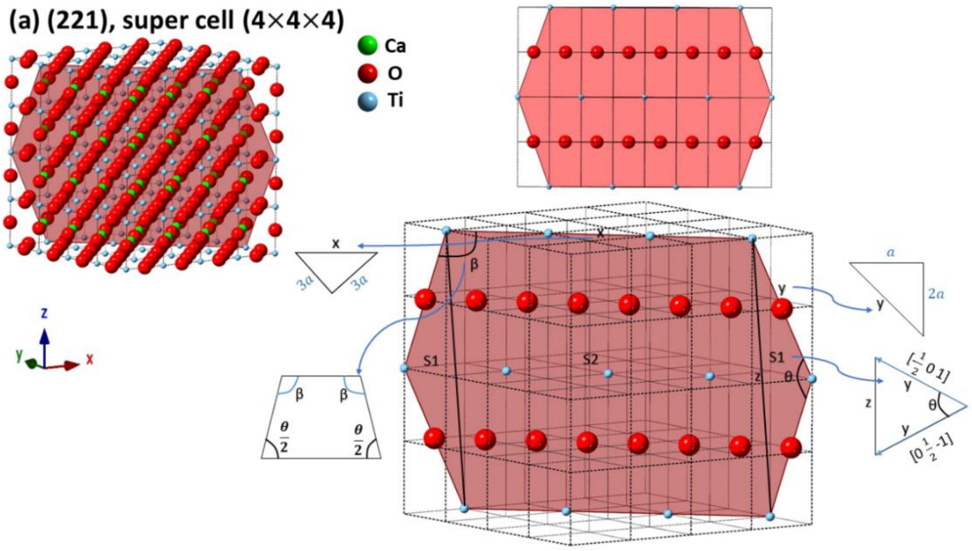

$$(x)^2 = (3a)^2 + (3a)^2 = (3 \times 3.79)^2 + (3 \times 3.79)^2 \longrightarrow x = 16.08$$

$$(y)^2 = (a)^2 + (2a)^2 = (3.79)^2 + (2 \times 3.79)^2 \longrightarrow y = 8.48$$

area of the plane (221): $S = 2 \times S1 + S2$

Angle between two miller cubic directions: $[u_1\ v_1\ w_1] < [u_2\ v_2\ w_2]$ $\left[0\ \frac{1}{2}\ -1\right] \measuredangle \left[-\frac{1}{2}\ 0\ 1\right]$

$$cos\theta = \frac{[u_1 u_2 + v_1 v_2 + w_1 w_2]}{\sqrt{(u_1^2 + v_1^2 + w_1^2)}\sqrt{(u_2^2 + v_2^2 + w_2^2)}} = \frac{-1}{\sqrt{\left(\frac{1}{4}+1\right) \times (\frac{1}{4}+1)}} = -0.8 \longrightarrow \theta = 143.13 \longrightarrow \beta = \left(\frac{360-(143.13)}{2}\right) = 108.44$$

$$S1 = \frac{1}{2} \times y \times y \times \sin\theta = \frac{1}{2} \times 8.48 \times 8.48 \times \sin(143.13) = 21.57$$

Cosines law: $z^2 = (y)^2 + (y)^2 - 2(y)(y)cos\theta$

$$z^2 = (8.48)^2 + (8.48)^2 - 2(8.48)(8.48)cos\theta \longrightarrow z = 16.08 \longrightarrow S2 = z \times x = 16.08 \times 16.08 = 258.57$$

$$\longrightarrow S = 258.57 + 2 \times 21.57 = 301.71$$

number of atoms in the plane (221) × area of each atom in the plane (221) =

$$\left[\left((2 \times \frac{143.13}{360} + 4 \times \frac{108.44}{360} + 4 \times \frac{1}{2} + 3) \times \pi\left(r_{Ti^{4+}}\right)^2\right) + \left((12 + 4 \times \frac{1}{2}) \times \pi\left(r_{O^{2-}}\right)^2\right)\right] = [(7 \times \pi\,(0.60)^2) + (14 \times \pi\,(1.40)^2)] = 94.12$$

$$\text{Planar density} = \frac{\text{number of atoms in the plane (221)} \times \text{area of each atom in the plane (221)}}{\text{area of the plane (221)}} = \frac{94.12}{301.71} = 0.31$$

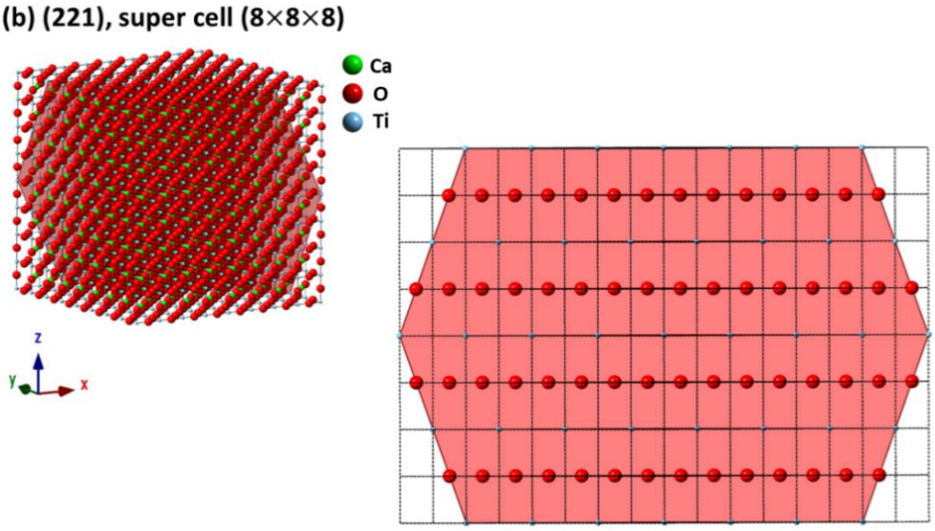

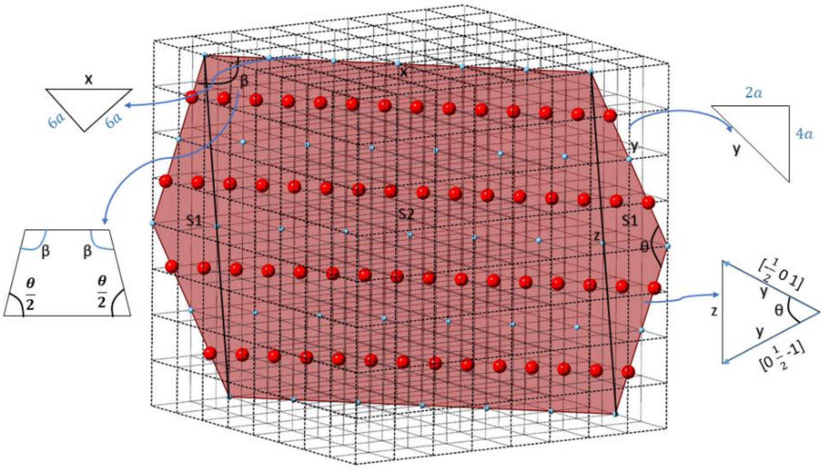

$(x)^2 = (6a)^2 + (6a)^2 = (6 \times 3.79)^2 + (6 \times 3.79)^2 \longrightarrow x = 32.16$

$(y)^2 = (2a)^2 + (4a)^2 = (2 \times 3.79)^2 + (4 \times 3.79)^2 \longrightarrow y = 16.95$

area of the plane (221): $S = 2 \times S1 + S2$

Angle between two miller cubic directions: $[u_1\ v_1\ w_1] < [u_2\ v_2\ w_2]$ $\left[0\ \frac{1}{2}\ -1\right] \angle \left[-\frac{1}{2}\ 0\ 1\right]$

$$cos\theta = \frac{[u_1u_2 + v_1v_2 + w_1w_2]}{\sqrt{(u_1^2 + v_1^2 + w_1^2)}\sqrt{(u_2^2 + v_2^2 + w_2^2)}} = \frac{-1}{\sqrt{\left(\frac{1}{4}+1\right) \times (\frac{1}{4}+1)}} = -0.8 \longrightarrow \theta = 143.13 \longrightarrow \beta = \left(\frac{360 - (143.13)}{2}\right) = 108.44$$

$S1 = \frac{1}{2} \times y \times y \times \sin\theta = \frac{1}{2} \times 16.95 \times 16.95 \times \sin(143.13) = 86.19$ Cosines law: $z^2 = (y)^2 + (y)^2 - 2(y)(y)cos\theta$

$z^2 = (16.95)^2 + (16.95)^2 - 2(16.95)(16.95)cos\theta \longrightarrow z = 32.16 \longrightarrow S2 = z \times x = 32.16 \times 32.16 = 1034.27$

$\longrightarrow S = 1034.27 + 2 \times 86.19 = 1206.65$

number of atoms in the plane (221) × area of each atom in the plane (221) =

$$\left[\left((2 \times \frac{143.13}{360} + 4 \times \frac{108.44}{360} + 14 \times \frac{1}{2} + 19) \times \pi\,(r_{Ti^{4+}})^2\right) + \left((52 + 8 \times \frac{1}{2}) \times \pi\,(r_{O^{2-}})^2\right)\right] = [(28 \times \pi\,(0.60)^2) + (56 \times \pi\,(1.40)^2)] = 376.49$$

$$\text{Planar density} = \frac{\text{number of atoms in the plane (221)} \times \text{area of each atom in the plane (221)}}{\text{area of the plane (221)}} = \frac{376.49}{1206.65} = 0.31$$

Figure S8. Geometry of planes and calculations of planar density of (a) (221) super cell (4×4×4) and (b) (221) super cell (8×8×8).

(a) (311), super cell (3×3×3)

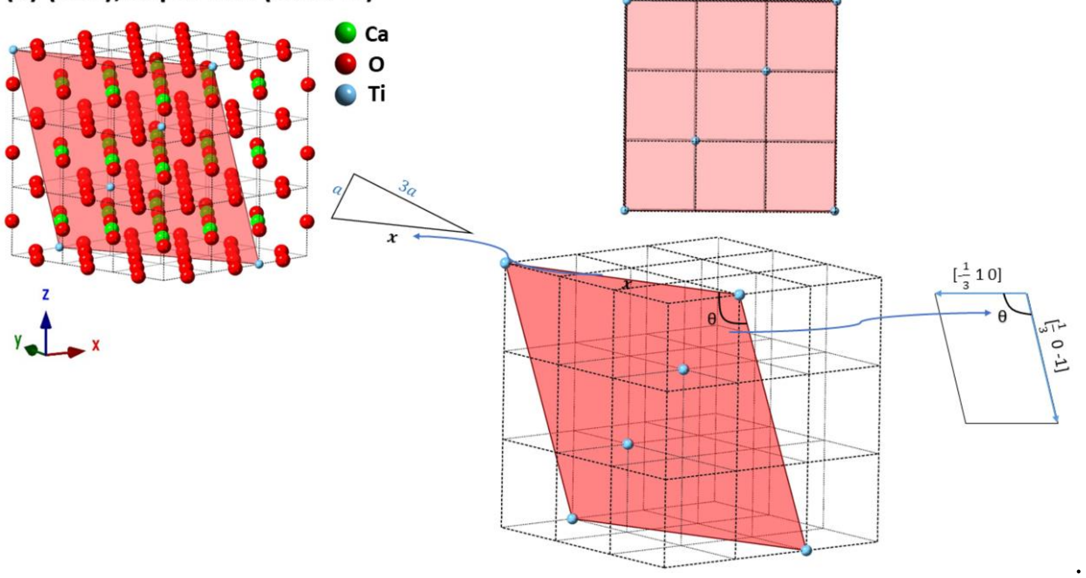

$$(x)^2 = (a)^2 + (3a)^2 = (3.79)^2 + (11.37)^2 \longrightarrow \quad x = 11.99$$

Angle between two miller cubic directions: $[u_1\ v_1\ w_1] < [u_2\ v_2\ w_2]$ $\left[-\frac{1}{3}\ 1\ \ 0\right] \angle \left[\frac{1}{3}\ 0\ -1\right]$

$$cos\theta = \frac{[u_1u_2 + v_1v_2 + w_1w_2]}{\sqrt{(u_1^2+v_1^2+w_1^2)}\sqrt{(u_2^2+v_2^2+w_2^2)}} = \frac{-\frac{1}{9}}{\sqrt{\left(\frac{1}{9}+1\right)\times(\frac{1}{9}+1)}} = -0.1 \longrightarrow \quad \theta = 95.74$$

area of the plane (311): $S = x \times x \times \sin\theta = 11.99 \times 11.99 \times \sin 95.74 = 143.04$

number of atoms in the plane (311) × area of each atom in the plane (311) =

$$\left[\left((2 \times \frac{95.74}{360} + 2 \times \frac{84.26}{360} + 2) \times \pi \left(r_{Ti^{4+}}\right)^2\right)\right] = (3 \times \pi \times (0.6)^2) = 3.39$$

$$\text{Planar density} = \frac{\text{number of atoms in the plane (311)} \times \text{area of each atom in the plane (311)}}{\text{area of the plane (311)}} = \frac{3.39}{143.04} = 0.02$$

(b) (311), super cell (4×4×4)

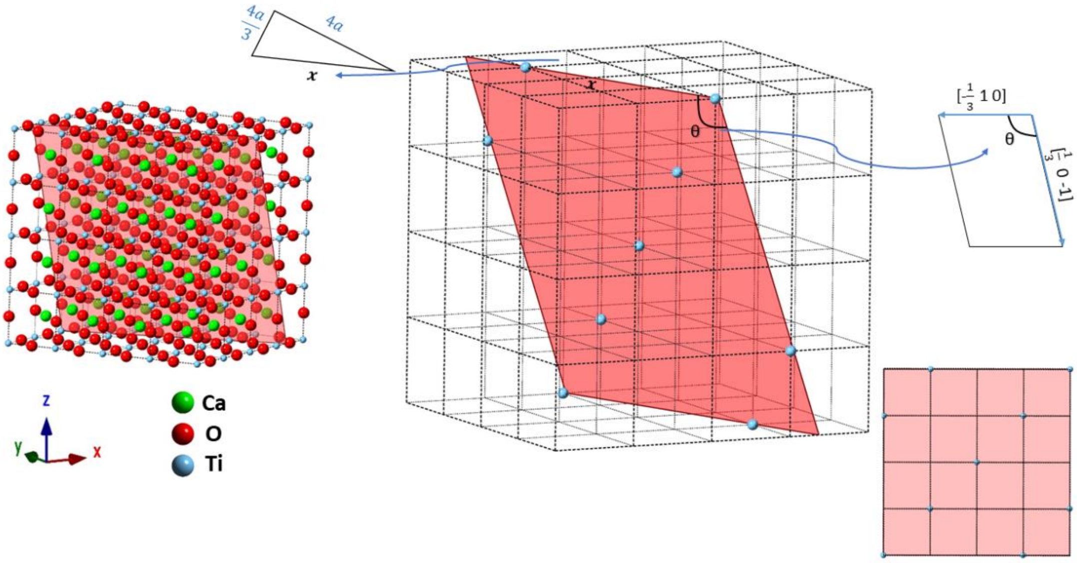

$$(x)^2 = \left(\frac{4a}{3}\right)^2 + (4a)^2 = (5.05)^2 + (15.16)^2 \longrightarrow x = 15.99$$

Angle between two miller cubic directions: $[u_1\, v_1\, w_1] < [u_2\, v_2\, w_2]$ $\left[-\frac{1}{3}\ 1\ 0\right] \angle \left[\frac{1}{3}\ 0\ -1\right]$

$$cos\theta = \frac{[u_1u_2 + v_1v_2 + w_1w_2]}{\sqrt{(u_1^2 + v_1^2 + w_1^2)}\,\sqrt{(u_2^2 + v_2^2 + w_2^2)}} = \frac{-\frac{1}{9}}{\sqrt{\left(\frac{1}{9}+1\right) \times \left(\frac{1}{9}+1\right)}} = -0.1 \longrightarrow \theta = 95.74$$

area of the plane (311): $S = x \times x \times \sin\theta = 15.99 \times 15.99 \times \sin 95.74 = 254.40$

number of atoms in the plane (311) × area of each atom in the plane (311) =

$$\left[\left((2 \times \frac{95.74}{360} + 4 \times \frac{1}{2} + 3) \times \pi\left(r_{Ti^{4+}}\right)^2\right)\right] = (5.53 \times \pi \times (0.6)^2) = 6.25$$

Planar density $= \frac{\text{number of atoms in the plane (311)} \times \text{area of each atom in the plane (311)}}{\text{area of the plane (311)}} = \frac{6.25}{254.40} = 0.02$.

(c) (311), super cell (8×8×8)

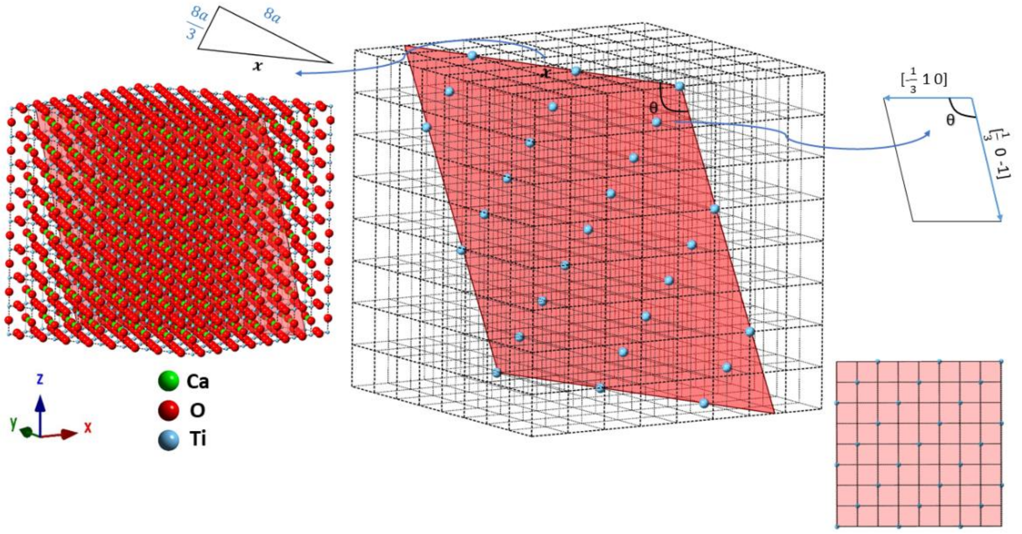

$$(x)^2 = (\frac{8a}{3})^2 + (8a)^2 = (10.11)^2 + (30.32)^2 \longrightarrow x = 31.96$$

Angle between two miller cubic directions: $[u_1\, v_1\, w_1] < [u_2\, v_2\, w_2]$ $\left[-\frac{1}{3}\ 1\ 0\right] < \left[\frac{1}{3}\ 0\ -1\right]$

$$cos\theta = \frac{[u_1u_2 + v_1v_2 + w_1w_2]}{\sqrt{(u_1^2 + v_1^2 + w_1^2)}\sqrt{(u_2^2 + v_2^2 + w_2^2)}} = \frac{-\frac{1}{9}}{\sqrt{\left(\frac{1}{9}+1\right) \times (\frac{1}{9}+1)}} = -0.1 \longrightarrow \theta = 95.74$$

$$\text{area of the plane (311): } S = x \times x \times \sin\theta = 31.96 \times 31.96 \times \sin 95.74 = 1016.32$$

number of atoms in the plane (311) × area of each atom in the plane (311) =

$$\left[\left((2 \times \frac{95.74}{360} + 8 \times \frac{1}{2} + 17) \times \pi\,(r_{Ti^{4+}})^2\right)\right] = (21.53 \times \pi \times (0.6)^2) = 24.35$$

$$\text{Planar density} = \frac{\text{number of atoms in the plane (311)} \times \text{area of each atom in the plane (311)}}{\text{area of the plane (311)}} = \frac{24.35}{1016.32} = 0.02$$

Figure S9. Geometry of planes and calculations of planar density of (a) (311) super cell (3×3×3), (b) (311) super cell (4×4×4) and (c) (311) super cell (8×8×8).

(a) (222), super cell (3×3×3)

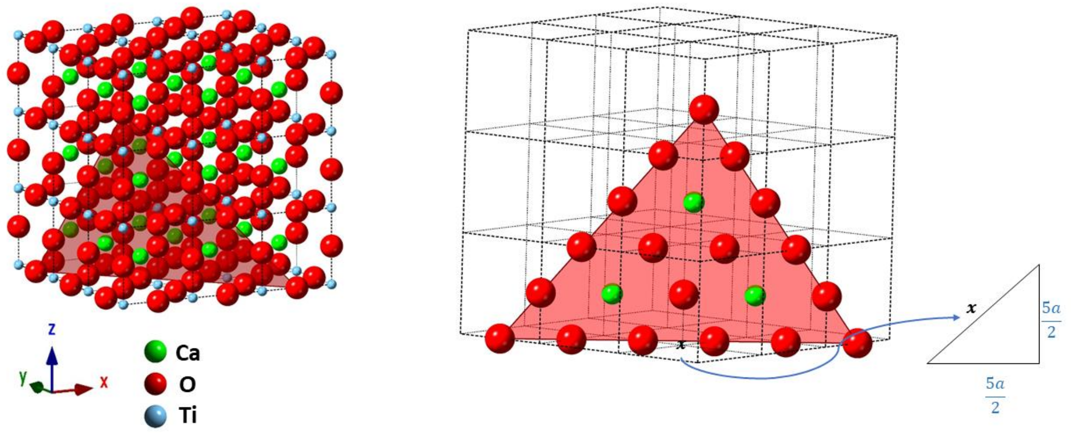

$$(x)^2 = (5\frac{a}{2})^2 + (5\frac{a}{2})^2 = (5 \times 1.89)^2 + (5 \times 1.89)^2 \longrightarrow x = 13.36$$

$$\text{area of the plane (222): } S = 0.5 \times x \times x \times \sin 60 = 0.5 \times 13.36 \times 13.36 \times 0.866 = 77.28$$

number of atoms in the plane (222) × area of each atom in the plane (222) = $\left[\left((3 \times \frac{1}{6} + 12 \times \frac{1}{2} + 3) \times \pi\,(r_{O^{2-}})^2\right)\right] + \left[\left(3 \times \pi \times (r_{Ca^{+}})^2\right)\right] =$

$$[(9.5 \times \pi\,(1.40)^2)] + [(3 \times \pi\,(1)^2)] = 67.92$$

$$\text{Planar density} = \frac{\text{number of atoms in the plane (222)} \times \text{area of each atom in the plane (222)}}{\text{area of the plane (222)}} = \frac{67.92}{77.28} = 0.88$$

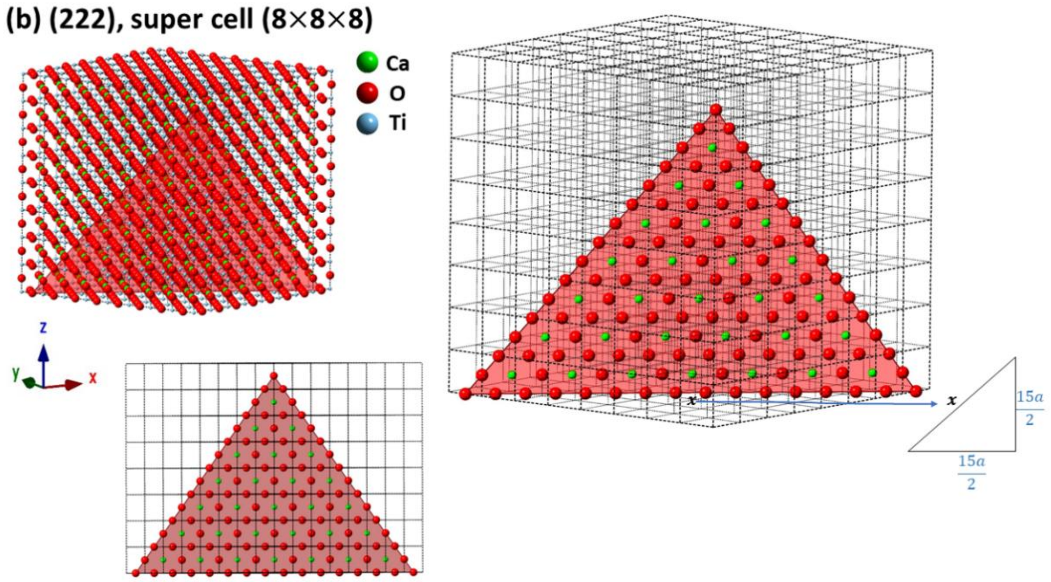

$$(x)^2 = (15\frac{a}{2})^2 + (15\frac{a}{2})^2 = (15 \times 1.89)^2 + (15 \times 1.89)^2 \longrightarrow x = 40.09$$

area of the plane (222): $S = 0.5\times x \times x \times \sin 60 = 0.5 \times 40.09 \times 40.09 \times 0.866 = 695.92$

number of atoms in the plane (222) × area of each atom in the plane (222) $= \left[\left((3 \times \frac{1}{6} + 42 \times \frac{1}{2} + 63) \times \pi \left(r_{O^{2-}}\right)^2\right)\right] + \left[\left(28 \times \pi \times \left(r_{Ca^{+}}\right)^2\right)\right] =$

$[(84.5 \times \pi\ (1.40)^2)] + [(28 \times \pi\ (1)^2)] = 608.28$

$$\text{Planar density} = \frac{\text{number of atoms in the plane (222)} \times \text{area of each atom in the plane (222)}}{\text{area of the plane (222)}} = \frac{608.28}{695.92} = 0.88$$

Figure S10. Geometry of planes and calculations of planar density of (a) (222) super cell (3×3×3), (b) (222) super cell (8×8×8).

References

[1] Rabiei M, Palevicius A, Monshi A, Nasiri S, Vilkauskas A and Janusas G 2020 Comparing Methods for Calculating Nano Crystal Size of Natural Hydroxyapatite Using X-Ray Diffraction *Nanomaterials* **10** 1627

[2] Shahidi M M, Ehsani M H, Rezagholipour Dizaji H and Ghazi M E 2020 Effect of silver, gold, and platinum substrates on structural and optical properties of tilted nanocolumnar SnS films *J. Mater. Sci. Mater. Electron.* **31** 2030–9
